# Orally administrable photo-crosslinked coumaroyl-modified gelatin-CNC nanofibers for ulcerative colitis environment-triggered release of 5-aminosalicylic acid

**DOI:** 10.1186/s12951-026-04889-y

**Published:** 2026-08-12

**Authors:** Jomon George Joy, Garima Sharma, Mydhili Govindarasu, Panalee Pomseethong, Songrae Kim, Jin-Chul Kim

**Affiliations:** 1 Department of Biomedical Science & Institute of Bioscience and Biotechnology, Kangwon National University, Chuncheon, 24341 Republic of Korea; 2 Institute of Forest Science, Kangwon National University, Chuncheon, 24341 Republic of Korea; 3 Metropolitan Seoul Center Korea Basic Science Institute (KBSI), Seoul, 02841 Republic of Korea

**Keywords:** Ulcerative Colitis, Photo-crosslinked, Orally administrable nanofibers, Sulfatase enzyme, Controlled release

## Abstract

**Background:**

Conventional ulcerative colitis (UC) therapies are limited by low colonic bioavailability and systemic side effects due to premature drug release in the upper gastrointestinal tract. To overcome these issues, we developed an orally administrable, photo-crosslinked nanofiber composed of coumaroyl-modified gelatin (Cm-Gel) and coumaroyl-modified cellulose nanocrystals (Cm-CNC) for colon-targeted delivery of 5-aminosalicylic acid (5-ASA).

**Results:**

The nanofibers was fabricated by electrospinning a Cm-Gel: Cm-CNC composition followed by UV-induced photodimerization, forming a stable photo-crosslinked network. Spectroscopic and microscopic analyses confirmed the electrostatic interactions between Cm-Gel and Cm-CNC, as well as the successful photodimerization of coumaroyl groups. The Cm-Gel: Cm-CNC nanofibers exhibited minimal 5-ASA release under acidic conditions, while release was markedly enhanced under colonic pH (6.5–7.5). This release was further enhanced in the presence of sulfatase enzyme (a colonic enzyme) due to network dissociation and triggered on-demand drug release. This was attributed to the incorporation of Cm-CNC, which significantly improved mechanical strength, network integrity, and swelling capacity, contributing to sustained and controlled release of the payload due to photodimerization. At non-cytotoxic doses, the calcein-loaded Cm-Gel: Cm-CNC nanofibers showed significantly higher cellular uptake than calcein-loaded Cm-Gel only nanofibers. Intracellular trafficking studies further demonstrated effective lysosomal localization of the nanofibers, while DCFH-DA analysis confirmed significant reduction of intracellular oxidative stress, and hemolysis testing verified excellent hemocompatibility. In a DSS-induced colitis mouse model, in vivo fluorescence imaging revealed that the DiR-loaded Cm-Gel: Cm-CNC nanofibers achieved significantly higher intestinal uptake compared with free DiR alone. Similarly, 5-ASA-loaded nanofibers effectively alleviated DSS-induced colonic inflammation, reduced histopathological damage as confirmed by H&E staining and tight-junction protein (Occludin, Claudin-1, and ZO-1) immunohistochemical staining, and suppressed pro-inflammatory cytokines (IL-6 and TNF-α) in the blood serum. These therapeutic outcomes surpassed those of free 5-ASA and unloaded nanofibers.

**Conclusions:**

The orally administrable photo-crosslinked Cm-Gel: Cm-CNC nanofibers provides complete biodegradability and targeted, sulfatase enzyme- and pH-responsive release of 5-ASA in the colon. Its strong anti-inflammatory efficacy, biocompatibility, and controlled-release behaviour highlight its promise as a safe, colon-specific, multi-stimuli-responsive drug-delivery platform for ulcerative colitis.

**Graphical Abstract:**

**Figure:**
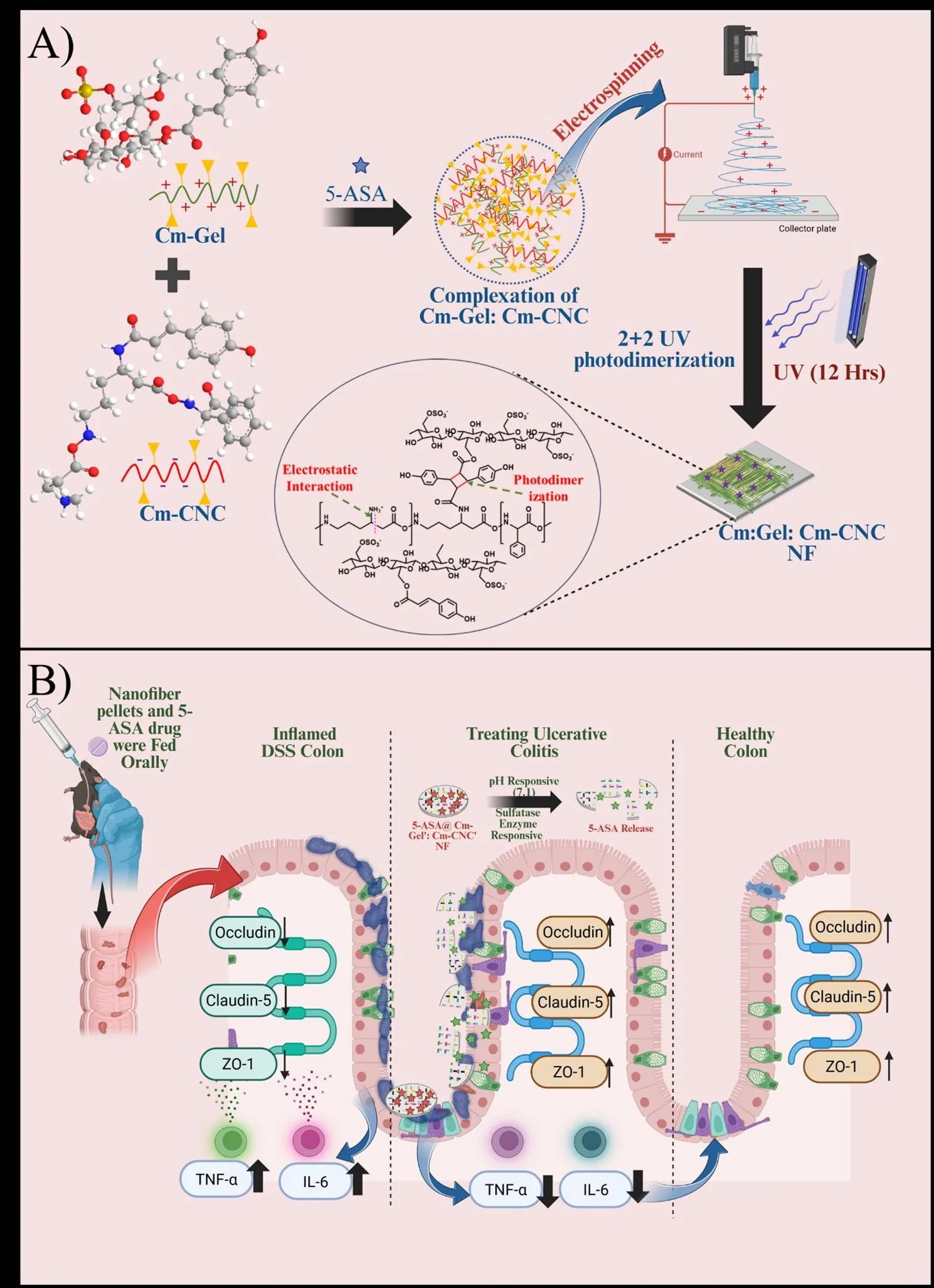

**Supplementary Information:**

The online version contains supplementary material available at https://doi.org/10.1186/s12951-026-04889-y.

## Introduction

Ulcerative colitis (UC), a subtype of inflammatory bowel disease (IBD), is a chronic, relapsing condition characterized by inflammation and ulceration of the colonic mucosa [1, 2]. Despite significant advancements in UC management, its treatment remains a major clinical challenge due to the limited targeting specificity of conventional oral therapeutics and their undesirable systemic side effects [3–5]. The heterogeneity of disease manifestation, the variability of drug absorption along the gastrointestinal (GI) tract, and the chronic nature of UC further complicate long-term therapy and patient adherence [3, 4, 6–8]. A growing need exists for advanced drug delivery systems that can precisely deliver therapeutic agents to inflamed colonic sites with minimal systemic exposure.

To overcome these challenges, stimuli-responsive nanocarriers have emerged as promising tools for site-specific drug delivery in UC and other GI diseases [9–12]. These systems are engineered to release their cargo in response to intrinsic pathological signals, such as pH variation, enzymatic activity, oxidative stress, or microbial metabolites, which are often elevated at inflammation sites. Among these triggers, pH-responsive carriers have gained significant traction due to the well-established pH gradient along the GI tract (from pH 1–3 in the stomach to pH 6–7.5 in the colon), enabling site-specific drug release and protection from early degradation [11–14].

However, pH-sensitive systems alone may lack precision, especially in cases where pH shifts are insufficient to distinguish between healthy and diseased colon tissues. Moreover, their efficacy is often limited by premature release in the small intestine or by inter individual variations in GI pH [10–12]. Therefore, integrating more disease-specific stimuli, such as enzymatic Sulfatases are hydrolytic enzymes involved in the removal of sulfate groups from sulfated substrates, and their expression is markedly elevated in the inflamed colon during UC [15–17]. These enzymes are secreted by infiltrating immune cells, damaged epithelial cells, and certain gut microbiota such as Bacteroides and Clostridia, all of which are upregulated during inflammatory flares [18–20]. Unlike pH or microbial population shifts that can be non-specific or dynamic, sulfatase expression correlates more directly with inflammation severity and localization. As such, sulfatase represents an ideal molecular target for enzyme-triggered drug release, ensuring therapeutic activation only at sites of active disease [20, 21]. Despite their promise, sulfatase-responsive systems remain underutilized in oral drug delivery due to the limited availability of safe, sulfatase-degradable materials. Few studies have developed fully biocompatible, food-grade formulations suitable for long-term, oral use, especially in sensitive groups like pediatric UC patients, offering a selective and robust strategy for on-demand drug release.

Electrospun nanofibers have recently gained attention as versatile drug carriers, particularly for mucosal and GI applications [22–24]. Compared to traditional nanocarriers such as liposomes, polymeric micelles, and hydrogels, nanofibers offer several distinct advantages: (i) high surface-area-to-volume ratio [25], (ii) superior drug-loading capacity [26], (iii) tunable porosity and mechanical strength [27, 28], and (iv) the ability to encapsulate both hydrophilic and hydrophobic drugs [29–31]. When fabricated using orally administrable and biodegradable materials such as gelatin and cellulose nanocrystals (CNCs), these fibers become especially attractive for chronic oral therapy [32].

**Scheme 1:**
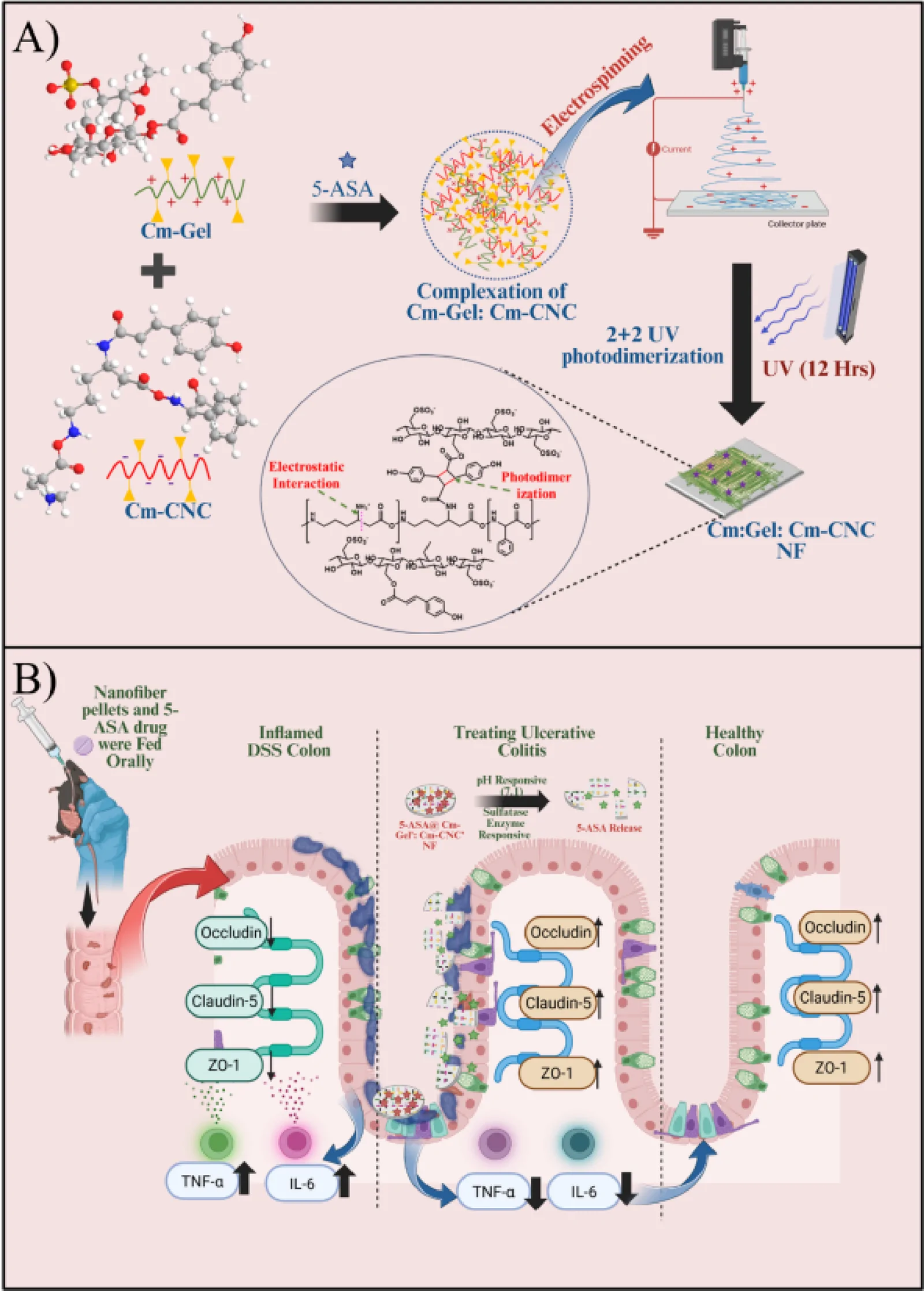
**A**) Schematic representation of the electrospun nanofibers from Cm-Gel and Cm-CNC and their sulfatase and pH-responsive release. **B**) Schematic representation of the treatment of ulcerative colitis using the 5-ASA loaded Cm-Gel and Cm-CNC nanofibers. (Created using Bio render)

In this study, we present a novel dual-responsive, orally administrable nanofibers for colon-targeted delivery of 5-aminosalicylic acid (5-ASA) in UC therapy. Orally administered 5-ASA is rapidly absorbed in the upper intestine, causing systemic side effects and reducing its therapeutic concentration in the colon. For UC, a variety of oral delivery methods have been developed, such as mucoadhesive nanoparticle [33], pH-responsive hydrogels [3, 34], polysaccharide-based carriers [35], and microbiota-degradable [36] matrices. Although these platforms can protect drugs from harsh gastrointestinal pH and enzymatic degradation, important clinical limitations remain. This is possibly due to relying on the single physiological stimuli-based drug release platform which results in early drug release or inadequate selectivity towards inflammatory colonic regions. Furthermore, certain formulations show poor cellular contact, limited mucosal retention, or insufficient structural stability under gastrointestinal conditions [37, 38]. Therefore, multifunctional delivery systems that can combine controlled release, extended retention, selective colon targeting, and improved therapeutic interaction with the intestinal epithelium are prerequisite. To address this, we developed an electrospun nanofibers composed of coumaroyl-modified gelatin (Cm-Gel) and sulfated cellulose nanocrystals (Cm-CNC), stabilized via UV-induced coumaroyl dimerization (Scheme 1 A). The purpose of UV treatment in this system is solely to induce crosslink formation within the nanofibers network, thereby improving structural integrity, mechanical stability, and controlled drug release behavior. Sulfated CNCs enable enzymatic release triggered by colonic sulfatases, while the gelatin matrix exhibits pH-responsive swelling (pH 6.5–7.5), promoting site-specific drug diffusion [15, 17, 20]. Made entirely from orally administrable, biodegradable materials, this system ensures safety and eliminates the need for synthetic carriers. Its mucoadhesive properties enhance colonic retention, and the oral feeding route, mimicking dietary intake, improves compliance, particularly in pediatric or chronic cases [39–43]. The nanofibers support high drug loading and potential co-delivery of multiple agents for comprehensive UC treatment. We report its synthesis, characterization, and therapeutic efficacy in a DSS-induced colitis model (Scheme 1B). To our knowledge, this is the first sulfatase- and pH-responsive orally administrable nanofiber enabling enzyme-triggered UC therapy via oral feeding, offering a safe, non-invasive strategy with broader potential for mucosal inflammatory diseases.

## Experimental section

### Materials

Gelatin-b, Coumaric acid (Cm), N-Hydroxysuccinamide (NHS), Sodium Hydroxide (NaOH) was purchased from Sigma-Aldrich. 1-(3-Dimethylaminopropyl)−3-ethylcarbodiimide Hydrochloride (EDC), 4-Dimethylaminopyridine (DMAP), Dimethylformamide (DMF), Triethylamine (TEA), Dimethyl sulfoxide-d6 (DMSO-d6) 99.9 atom % D, contains 0.03%v/v TMS, Dimethyl sulfoxide anhydrous (DMSO) was obtained from Daejung Chemical & Metals Co., Ltd. (Korea). N, N`-dicyclohexylcarbodiimide (DCC) was purchased from Tokyo Chemical Industry Co., Ltd. (Tokyo, Japan). Cellulose nanocrystal (CNC) was obtained from Celluforce Inc. (Quebec, Canada)). sulfatase from Helix pomatia and Dextran sulfate sodium salt (DSS, MW 4 104) were purchased from Sigma-Aldrich Co. (St. Louis, MO, USA). DiR (1,10-dioctadecyl-3,3,30,30-tetramethyl indotricarbocyanine iodide) was obtained from Thermo Fisher Scientific.HistoChoice^®^ clearing agent and DPX mountant for histology were purchased from Sigma-Aldrich. Mouse IL-6 ELISA kit and Mouse TNF alpha High sensitivity ELISA kit were purchased from Invitrogen by Thermo Scientific. All chemicals were purchased in analytical grade.

### Synthesis and characterization of Cm-Gel and Cm-CNC

Coumaroyl-modified gelatin (Cm-Gel) was synthesized via a carbodiimide-mediated conjugation process. Specifically, 0.612 mmol of coumaric acid (Cm) was dissolved in 1 N sodium hydroxide, and the pH was adjusted to 8.0 using 1 N hydrochloric acid. To activate the carboxyl group of Cm, 1.64 mmol of EDC was added under ice-cold conditions (4 °C) and maintained for 1 h. In parallel, 1 g of gelatin B was dissolved in 10 mL of phosphate-buffered saline (PBS, 30 mM, pH 7.4). The gelatin solution was then added to the activated Cm mixture and stirred continuously at 30 °C overnight to facilitate covalent coupling. After the reaction, the mixture was subjected to dialysis (MWCO 3500 Da) against distilled water for 72 h and subsequently lyophilized to yield the Cm-Gel product.

For the preparation of coumaroyl-modified cellulose nanocrystals (Cm-CNC), 2 g of CNCs were dispersed in 80 mL of dimethylformamide (DMF) using a tip sonicator (VC505, SONICS, USA) for 10 min, operating in 10-second on/off cycles. The dispersion was cooled in an ice bath, followed by the addition of 2.85 mL triethylamine (TEA) and 1.5 g 4-dimethylaminopyridine (DMAP). Separately, a known amount of Cm was dissolved in 10 mL of DMF and added dropwise to the CNC dispersion under continuous stirring. The esterification reaction proceeded for 12 h at ambient temperature. The resulting product was purified by centrifugation (4000 rpm for 10 min) with tetrahydrofuran (THF) three times, followed by three washes with distilled water using the same method. The purified Cm-CNC was then freeze-dried for storage and further use.

All materials, Gelatin, Cm-Gel, CNC, and Cm-CNC, were dried in a vacuum oven to remove residual moisture. For structural characterization, Gel and Cm-Gel were dissolved in deuterium oxide (D₂O), while CNC and Cm-CNC were dissolved in deuterated DMSO (DMSO-d₆), and analyzed using proton nuclear magnetic resonance (^1^H-NMR) spectroscopy (Bruker Avance 400 MHz, Karlsruhe, Germany). Fourier-transform infrared (FT-IR) spectroscopy was also performed using a PerkinElmer Frontier spectrophotometer to confirm functional group modifications.

The coumaroyl content of Cm-Gel and Cm-CNC was quantified by UV-Vis spectrophotometry (JENWAY 6505, UK). A calibration curve was generated using Cm standards prepared in 10 mM HEPES buffer (for Cm-Gel, concentration range: 0.000122–0.25 mg/mL) and in DMSO (for Cm-CNC, range: 0.0078125–0.5 mg/mL). Absorbance measurements were recorded at 280 nm to calculate coumaroyl content based on the corresponding standard curves.

To investigate the crystalline structure of CNC before and after modification, X-ray diffraction (XRD) analysis was performed using an X’Pert PRO MPD diffractometer (Malvern Panalytical). The measurements employed Cu Kα radiation with wavelengths λ = 1.5406 Å (Kα₁) and λ = 1.5444 Å (Kα₂), using a weighted average of 1.5418 Å. Scans were recorded across a 2θ range of 5°–45°, with a step size of 0.01313°, a divergence slit of 1.52 nm, and an operating voltage of 40 kV.

### Complex formation of Cm-Gel and Cm-CNC

To evaluate the potential for complex formation between Cm-Gel and Cm-CNC, the two components were combined in various mass ratios within an acetic acid: H_2_O solvent system. Solutions of Cm-Gel and Cm-CNC were each prepared at a concentration of 2% (w/v) and mixed in different proportions: 9:1, 8:2, 7:3, 6:4, 5:5, 4:6, 3:7, 2:8, and 1:9 (Cm-Gel: Cm-CNC). The mixtures were placed on a roller mixer and stirred continuously for 24 hs to ensure complete complexation.

The formation of complexes was confirmed by dynamic light scattering (DLS), which was used to assess particle size and light scattering intensity. Fourier-transform infrared (FT-IR) spectroscopy was employed to investigate the electrostatic interactions between the components within the complexes. To facilitate imaging and colocalization studies, Cm-Gel was fluorescently labeled with fluorescein isothiocyanate (FITC), and Cm-CNC was labeled with rhodamine B isothiocyanate (RBITC). Complexes with Cm-Gel: Cm-CNC mass ratios of 9:1, 8:2, and 7:3 were subjected to confocal laser scanning microscopy to visualize complex formation. Transmission electron microscopy (TEM) was further used to examine the morphological characteristics of the formed complexes.

### Preparation of nanofibers

Electrospun nanofibers were fabricated using a mixture of Cm-Gel (20% w/v) and varying concentrations of Cm-CNC, with the maximum dispersible concentration of Cm-CNC being approximately 3% (w/v). Initially, Cm-CNC was dispersed in an acetic acid: H_2_O solvent system at concentrations of 1%, 2%, and 3% (w/v). Cm-Gel (20% w/v) was subsequently added to achieve final mass ratios of Cm-Gel: Cm-CNC as 9:1, 8.6:1.4, and 8:2. The mixtures were stirred overnight to ensure uniform blending and potential complex formation. The fiber-forming solutions were loaded into 5 mL syringes fitted with 25-gauge needles and processed using an electrospinning apparatus (NanpNC Co., Ltd., Seoul, Republic of Korea). Electrospinning was performed at a flow rate of 5 µL/min with an applied voltage of 18 kV/cm. A flat plate collector covered with aluminum foil was used to facilitate the easy removal of the fibers. As controls, nanofibers composed solely of gelatin and Cm-Gel were also prepared.

The resulting fibers included: Gelatin nanofiber (Gel NF), Cm-Gel nanofiber (Cm-Gel NF), Cm-Gel: Cm-CNC (9:1) NF, Cm-Gel: Cm-CNC (8.6:1.4) NF, Cm-Gel: Cm-CNC (8:2) NF. Following electrospinning, the fibers were air-dried at room temperature for 24 h. To induce photo-crosslinking through coumaroyl dimerization, the fibers were subsequently exposed to UV irradiation for 12 h.

For drug release studies, calcein-loaded nanofibers were prepared using the same electrospinning protocol. Calcein dye was incorporated into the Cm-Gel: Cm-CNC (8:2) matrix prior to electrospinning. Similarly, for therapeutic applications in ulcerative colitis, varying concentrations of 5-aminosalicylic acid (5-ASA) were loaded into the Cm-Gel: Cm-CNC (8:2) complex before fiber fabrication. All process parameters for electrospinning were maintained consistently throughout.

### Characterization of nanofibers

The fabricated nanofibers were first oven-dried and subsequently subjected to UV irradiation to induce photo-crosslinking via coumaroyl dimerization. Various analytical techniques were then employed to evaluate their structural, morphological, thermal, and physicochemical properties.

### FTIR and UV analysis

Fourier-transform infrared (FT-IR) spectroscopy with attenuated total reflectance (ATR) mode was used to investigate the chemical functional groups and electrostatic interactions within the nanofiber matrix. Prior to analysis, the fibers were ground into fine powder. Spectra were recorded using a Thermo Scientific FT-IR spectrophotometer (iN10/iS50). Nanofiber samples (1 × 1 cm) were immersed in PBS buffer (pH 7.4, 10 mM) and exposed to UV light for up to 6 h. Time-dependent UV absorbance spectra were recorded to monitor coumaroyl dimerization and confirm the extent of photo-crosslinking.

### Electron microscopy and confocal imaging

The surface morphology of the nanofibers was visualized using scanning electron microscopy (SEM; JEOL JSM-7900 F). Samples were sputter-coated with a thin layer of gold using a Leica EM ACE600 coater, and images were captured using a JEOL 6500 microscope. Elemental composition analysis was also performed to verify and quantify elements present in the fibers. To analysis the long term stability and morphological changes, the SEM images of the nanofiber Cm-Gel: Cm-CNC (8:2) was taken after 6 months which was compared with freshly prepared samples. Cm-Gel: Cm-CNC (8:2) nanofibers were sequentially incubated in simulated gastric fluid (SGF, pH 1.2), simulated intestinal fluid (SIF, pH 6.8), and simulated colonic fluid (SCF, pH 7.4) for 2 h each at 37 °C, followed by freeze-drying and SEM analysis to evaluate morphological changes. For confocal fluorescence microscopy (Carl Zeiss LSM900 with Airyscan2), FITC-labeled Cm-Gel and RBITC-labeled Cm-CNC were used to visualize the electrostatic interactions within the electrospun fibers. Additionally, nanofibers composed of Cm-Gel: Cm-CNC (8:2) and loaded with both a hydrophilic dye (RBITC) and a hydrophobic dye (Nile Red) were prepared. These were analyzed to confirm the co-encapsulation of different drug types within the nanofiber matrix.

### XRD, XPS, and thermal analysis

X-ray diffraction (XRD) analysis was conducted using a Panalytical X’Pert-Pro MPD diffractometer (Netherlands) with Cu Kα radiation to assess the crystallinity of the fibers incorporating Cm-CNC. Scans were performed over a 2θ range of 5° to 90° at a rate of 5 °C/min. The fibers were collected from aluminum foil, dried, and prepared for analysis. To evaluate sulfatase responsiveness, X-ray photoelectron spectroscopy (XPS) was carried out on CNC samples before and after treatment with sulfatase enzyme. Thermogravimetric analysis (TGA; TA Instruments SDT Q600, USA) was performed under a nitrogen atmosphere to determine the thermal stability of the fibers, using a heating rate of 10 °C/min from 30 °C to 700 °C.

### Mechanical strength, contact angle, and swelling behavior

Mechanical properties of various nanofibers, including Gel NF, Cm-Gel NF, and Cm-Gel: Cm-CNC (x: y) formulations, were evaluated using a universal testing machine (UTM; MCT-1150, AND Inc., Japan) operated at a constant crosshead speed of 10 mm/min. Cm-Gel and Cm-Gel: Cm-CNC (8:2) NF were stored under ambient conditions over 6 months, after which long term stability was evaluated by comparing mechanical properties. To assess surface hydrophilicity, contact angle measurements were performed by depositing deionized water droplets onto the fiber surfaces and analyzing them using a contact angle goniometer (Phoenix-MT). Swelling behavior was examined by cutting the nanofibers into 1 × 1 cm squares and immersing them in 1× PBS at room temperature. At designated time intervals, samples were removed, blotted gently with filter paper to eliminate surface water, weighed, and returned to the medium to monitor swelling kinetics over time.

### Drug release studies

#### Sulfatase- and pH-responsive release of calcein dye

To assess enzyme- and pH-triggered release behavior, calcein-loaded Cm-Gel: Cm-CNC (8:2) nanofibers were evaluated in both UV-crosslinked and non-crosslinked forms. The release experiments were conducted in phosphate-buffered saline (PBS) solutions with varying sulfatase concentrations (0, 20, 50, and 100 U/mL) and pH levels (2.1, 4.5, 5.5, and 7.0).

For each condition, 10 mL of sulfatase-containing PBS at the designated pH was placed in a 50 mL conical tube. A nanofiber mat (2 × 2 cm²) was immersed into the solution and incubated at room temperature with gentle shaking for 24 h. At predetermined intervals, 1 mL of the release medium was collected and replaced with fresh buffer to maintain the volume. Fluorescence intensity was measured using a fluorescence spectrophotometer (λ_ex = 495 nm, λ_em = 515 nm), and the cumulative release of calcein was calculated accordingly. Each condition was tested in triplicate (*n* = 3), and the same procedure was repeated for all pH levels.

#### Release of 5-aminosalicylic acid (5-ASA)

To investigate the therapeutic release profile, Cm-Gel: Cm-CNC (8:2) nanofibers encapsulating 5-aminosalicylic acid (5-ASA) were fabricated and subjected to UV-induced crosslinking. Fiber samples (2 × 2 mm²) were enclosed in dialysis bags (MWCO 3.5 kDa) and immersed in 20 mL of phosphate-buffered saline (PBS) at pH 2.1 or 7.0, either with or without 20 U/mL sulfatase enzyme. At designated time points, 1 mL of the release medium was collected and replaced with fresh PBS to maintain constant volume and sink conditions. Fluorescence spectroscopy was used to quantify the amount of released 5-ASA, utilizing the same excitation and emission parameters as described previously. All experiments were conducted in triplicate (n = 3), and cumulative drug release curves were plotted to evaluate the dual responsiveness to pH and sulfatase. For clarity, the Cm-Gel: Cm-CNC (8:2) nanofibers will henceforth be referred to as Cm-Gel’: Cm-CNC’ NF in subsequent sections.

### In vitro release study under simulated gastrointestinal conditions

The release behavior of 5-ASA from Cm-Gel’: Cm-CNC’ NF was evaluated sequentially under simulated gastrointestinal conditions to mimic oral administration [44, 45]. Briefly, nanofiber samples containing 5-ASA samples (2 × 2 mm^2^) were enclosed in dialysis bags (MWCO-3.5 kDa) and immersed in simulated gastric fluid (SGF, pH 1.2), simulated intestinal fluid (SIF, pH 6.8), and simulated colonic fluid (SCF, pH 7.4) with or without sulfatase, and incubated at 37 °C under gentle shaking. At predetermined time intervals, aliquots of the release medium were collected and replaced with an equal volume of fresh medium. The amount of released 5-ASA was quantified by fluorescence spectrophotometry, and the cumulative release percentage was calculated as a function of time.

### Release kinetic modeling

To elucidate the mechanism of drug release from the nanofibers, the release data obtained under different media conditions were fitted to kinetic models including the zero-order (Eq. 1),first-order (Eq. 2), Higuchi (Eq. 3), and Korsmeyer-Peppas (Eq. 4) models to determine the release kinetics of systems [39, 40]. A better-fit model was established using a correlation coefficient that was closest to one. Additionally, the obtained diffusional exponent “n” value is a sign of the mechanism that the drug release follows it. So, *n* ≤ 0.45, 0.45 < *n* > 1, and *n* = 1, and *n* > 1 indicate that the release is in Fickian diffusion, non-Fickian transport, case-II transport, and super case II transport mechanism mechanism respectively.

1 $$Q= K_ot$$

2 $$LnQ= LnQ_o-K_1t$$

3 $$Q = K_{H} t^{1/2}$$

4 $$LnQ=nLnt+Lnk_p$$

The rate constants of the zero-order, first-order, Higuchi, and Korsmeyer-Peppas kinetic models are referred to as k_0_, k_1_, k_H_, and k_P_ in (1), (2), (3), and (4). Additionally, the Q is the total amount of drugs released at a particular moment [39].

### Cell culture, cytotoxicity, and cellular interaction studies

Human colon epithelial carcinoma cells (CaCO-2) and murine macrophage cells (RAW 264.7) were employed to assess the cytocompatibility, cellular uptake, and immune response associated with the nanofiber formulations [4, 46, 47]. To evaluate cytotoxicity, a WST assay was conducted on both cell lines. Briefly, 5,000 cells per well were seeded into 96-well plates and allowed to adhere for 24 h at 37 °C in a humidified incubator with 5% CO₂. After incubation, the cells were treated with extraction solutions prepared from 1 × 1 cm² nanofiber samples (Cm-Gel NF and Cm-Gel’: Cm-CNC’ NF). Treatments were performed in triplicate (*n* = 3). After 24 h of exposure, cells were rinsed with PBS and incubated with WST reagent for 1 h. Absorbance was then measured at 450 nm to determine cell viability. To assess the direct effects of the fibers on cells, 1 × 1 cm² fiber samples were placed in 48-well plates. RAW 264.7 and CaCO-2 cells were seeded directly onto the fibers and incubated for 24 h. Following incubation, wells were rinsed with PBS, treated with WST reagent, and analyzed for viability as described above. Nanofibers loaded with 5-aminosalicylic acid (5-ASA@Cm-Gel’: Cm-CNC’ NF) were prepared at various drug concentrations (0.25%, 0.5%, and 1% w/v). These drug-loaded fibers were tested on CaCO-2 cells using the same viability protocol as described for the unloaded fibers. To visualize cellular uptake, confocal laser scanning microscopy was performed. Raw 264.7 cells were incubated with free calcein, calcein-loaded Cm-Gel NF, and calcein-loaded Cm-Gel’: Cm-CNC’ NF for up to 4 h. Fluorescent signals were analyzed to confirm internalization and cellular interaction of the nanofibers.

### Intracellular localization of nanofibers by LysoTracker Green and LAMP1 staining

RAW264.7 cells were seeded in 24-well plates containing sterile coverslips at a density of 1 × 10⁵ cells/well and cultured overnight to allow attachment. The cells were then treated with RBITC-labeled Cm-Gel′: Cm-CNC′ nanofibers, prepared from a 1 × 1 cm² nanofiber sample extract solution, for 6 h and 12 h.

For lysosomal membrane localization, the treated cells were fixed with 4% paraformaldehyde, permeabilized using 0.1% Triton X-100, and blocked with 1% bovine serum albumin (BSA). The cells were then incubated with a fluorophore-conjugated LAMP1 antibody Nuclei were counterstained with DAPI, and the samples were observed using confocal laser scanning microscopy. LAMP1 is a widely used lysosomal membrane marker for evaluating intracellular trafficking and lysosomal localization [48, 49].

For live-cell lysosomal staining, a separate set of treated cells was incubated with LysoTracker Green DND-26 in serum-free medium for 30 min at 37 °C in the dark. LysoTracker Green is a cell-permeable probe that accumulates in acidic organelles such as lysosomes (excitation/emission: 504/511 nm). After staining, cells were gently washed with PBS, counterstained with DAPI when required, and immediately imaged using confocal microscopy. The intracellular fate of the nanofibers was assessed by analyzing the colocalization of RBITC fluorescence with LAMP1 and LysoTracker Green signals in merged images at 6 h and 12 h (Cheng et al., 2018).

### Intestinal epithelial barrier function using CaCO-2 Transwell permeability assay

To evaluate intestinal epithelial barrier function, a Caco-2 monolayer model was established using a Transwell system (Costar Transwell, Millipore Corp., USA) with a polycarbonate membrane (pore size: 0.4 μm, surface area: 1.12 cm²). CaCO-2 cells were seeded at a density of 1 × 10⁵ cells/insert and cultured for 15 days to allow differentiation and tight junction formation, with the culture medium replaced every 2 days. The CaCO-2 Transwell model is widely used to mimic intestinal epithelial barrier function due to its ability to form polarized monolayers with functional tight junctions [16]. 

After monolayer formation, epithelial barrier dysfunction was induced by treating the cells with 3% (w/v) dextran sulfate sodium (DSS) for 24 h, which is known to disrupt tight junction integrity and increase paracellular permeability [50]. Following DSS exposure, the cells were treated with different formulations, including control (untreated), DSS-only, free 5-ASA, blank Cm-Gel’: Cm-CNC’ nanofibers, and 5-ASA-loaded Cm-Gel’: Cm-CNC’ nanofibers.

Paracellular permeability was assessed using FITC-dextran (4 kDa), a standard probe for evaluating epithelial barrier integrity. Briefly, FITC-dextran solution was added to the apical compartment, and after incubation, samples were collected from the basolateral compartment. The fluorescence intensity was measured using a microplate reader (excitation: 485 nm, emission: 520 nm). Increased fluorescence intensity indicates enhanced permeability and disruption of the epithelial barrier. All experiments were performed in triplicate (*n* = 3), and the data were expressed as mean ± standard deviation (SD) [16, 51].

### Intracellular ROS measurement by DCFH-DA assay and flow cytometry

Intracellular reactive oxygen species (ROS) generation was evaluated in RAW264.7 macrophages using 2′,7′-dichlorodihydrofluorescein diacetate (DCFH-DA), a cell-permeable fluorogenic probe commonly used to detect oxidative stress [52]. RAW264.7 cells were seeded in culture plates and allowed to adhere overnight. The cells were then stimulated with lipopolysaccharide (LPS) to induce an inflammatory oxidative-stress model for 12 h and treated with Cm-Gel′: Cm-CNC′ NF, free 5-ASA, or 5-ASA@Cm-Gel′: Cm-CNC′ NF, along with an untreated control group for 6 h. After the designated incubation period, the cells were washed with phosphate-buffered saline (PBS) and incubated with DCFH-DA in serum-free medium at 37 °C in the dark. Following dye loading, excess probe was removed by washing with PBS, and the cells were harvested for flow-cytometric analysis. Fluorescence from oxidized DCF was detected in the FITC channel, and intracellular ROS levels were quantified using the median fluorescence intensity (MFI) of the cell population. Data were obtained from three independent experiments and expressed as mean ± SD. Similar DCFH-DA flow-cytometric approaches have been widely used to quantify LPS-induced ROS production in RAW264.7 macrophages [52, 53].

### In vitro inflammatory response and cytokine analysis

The anti-inflammatory effect of the nanofibers was evaluated using a DSS-induced inflammatory model in CaCO-2 human colorectal adenocarcinoma cells [54, 55]. Briefly, CaCO-2 cells were seeded in culture plates and allowed to adhere overnight. To induce an inflammatory microenvironment, the cells were treated with dextran sulfate sodium (DSS, 3% w/v) for 24 h.

Following DSS stimulation, the cells were treated with different formulations, including control (untreated), DSS-only, free 5-ASA, blank Cm-Gel/Cm-CNC nanofibers, and 5-ASA-loaded Cm-Gel/Cm-CNC nanofibers. After an additional 24 h incubation, the culture supernatants were collected, and the levels of pro-inflammatory cytokines, including tumor necrosis factor-α (TNF-α) and interleukin-6 (IL-6), were quantified using enzyme-linked immunosorbent assay (ELISA) kits according to the manufacturer’s instructions. CaCO-2 cells treated with DSS alone were used as the positive inflammatory control, while untreated cells served as the negative control. All experiments were performed in triplicate (*n* = 3), and data were expressed as mean ± standard deviation (SD).

### Animal experiments

#### Establishment of experimental UC model and orally administrable nanofiber treatment

All animal experiments were conducted in accordance with protocols approved by the Animal Experiment Ethics Committee of Kangwon National University (KW-241105-4). UC was induced in C57BL/6 mice (6–8 weeks old, n = 5 per group) by administering 3% (w/v) dextran sulfate sodium (DSS) in their drinking water for seven consecutive days [8, 47]. Following one week of acclimation, mice were randomly divided into five groups: (1) untreated control, (2) DSS-only group, (3) DSS + Cm-Gel’: Cm-CNC’ NF (50 mg/kg), (4) DSS + free 5-ASA (3 mg/kg), and (5) DSS + 5-ASA-loaded Cm-Gel’: Cm-CNC’ NF (50 mg/kg containing 3 mg/kg 5-ASA). Treatment was administered via oral feeding in alternative days from day 8 to 14 following DSS induction. Prior to administration, nanofibers were dispersed in sterile distilled water at a concentration of 10 mg/mL and freshly prepared before each treatment. Mice were administered 100 µL of the suspension daily.

#### In vivo and ex vivo fluorescence imaging

To investigate colonic adhesion and distribution of nanofibers, DSS-induced and control mice were orally administered DiR-loaded Cm-Gel’: Cm-CNC’ NF or free DiR dye [56]. In vivo fluorescence imaging was performed at predetermined time points (0, 2, 4, 6, and 8 h) using an IVIS imaging system (VISQUE ^®^ InVivo ART 400 (Vieworks Co, Ltd., Anyang-si, Korea)). The fluorescence intensity of the tumor and major organs was quantified using the region-of-interest (ROI) analysis of the software provided by the manufacturer (Viewekrs Co, Ltd., Anyang-si, Korea). Mice were sacrificed at 8- and 24-h post-administration. Colons were collected without prior washing and imaged ex vivo to quantify total fluorescence intensity.

### Ex vivo mucoadhesion and mucus penetration studies

Freshly excised mouse colon tissues were opened longitudinally, gently rinsed with phosphate-buffered saline (PBS) to remove luminal contents while preserving the mucus layer, inverted to expose the mucosal surface, and fixed onto glass slides [57]. The tissues were then incubated with free DiR or DiR-loaded Cm-Gel’: Cm-CNC’ nanofiber dispersion for 1, 3, and 6 h. After incubation, the samples were gently washed with PBS to remove non-adherent material and immediately observed by confocal laser scanning microscopy to evaluate fluorescence retention and depth of penetration within the colonic mucosa. This ex vivo colonic adhesion approach was used to compare the mucosal interaction and retention behavior of the nanofiber formulation with that of free dye, as particle deposition in inflamed colonic mucus is strongly influenced by carrier–mucus interactions.

### Hemolysis assay

Fresh whole blood was collected from mice into anticoagulant-containing tubes, and red blood cells (RBCs) were isolated by centrifugation and washed several times with PBS to remove plasma and buffy coat [58]. The washed RBCs were then diluted in PBS to prepare an erythrocyte suspension for the hemolysis test. Free 5-ASA, 5-ASA-loaded nanofibers, PBS (negative control), and 1% Triton X-100 (positive control) were separately incubated with the RBC suspension at 37 °C for the designated period. After incubation, the samples were centrifuged, and the absorbance of the released hemoglobin in the supernatant was measured using a microplate reader. Hemolysis percentage was calculated using the standard equation [59].

$$Hemolysis (\%) = [(OD_{sample} - OD_{PBS})/ (OD_{Triton X-100} - OD_{PBS)}] \times 100$$

#### Evaluation of therapeutic efficacy in colitis mice

Therapeutic outcomes were assessed by monitoring clinical symptoms and inflammation in the DSS-induced UC model. The severity of colitis was assessed using the disease activity index (DAI), which was calculated based on body weight loss, stool consistency, and rectal bleeding. Body weight loss was scored as follows: 0 (no loss), 1 (1–5%), 2 (5–10%), 3 (10–15%), and 4 (> 15%). Stool consistency was graded as 0 (normal), 2 (loose stools), and 4 (diarrhea), while rectal bleeding was scored as 0 (no bleeding), 2 (occult blood positive), and 4 (gross bleeding). The final DAI score was calculated as the average of these three parameters: DAI = (weight loss score + stool consistency score + bleeding score)/3 [16]. After 7 days of treatment, mice were euthanized under CO₂ anesthesia. Colons were excised, rinsed with PBS, and measured for length, while spleens were weighed to assess systemic inflammation. For cytokine analysis, blood samples were collected from the retro-orbital sinus. Serum was separated by centrifugation at 3500 × g for 15 min, and the supernatant was analyzed for pro-inflammatory cytokines, including tumor necrosis factor-alpha (TNF-α) and interleukin-6 (IL-6), using ELISA.

### Histological and Immunohistochemical analysis of colon tissues

#### Hematoxylin and Eosin (H&E) staining

Colon samples from all groups were fixed in 10% formaldehyde, processed, and embedded in paraffin. Tissue Sect. (5 μm thick) were prepared using a microtome and mounted on silane-coated microscope slides. Slides were deparaffinized with Histo-clear, rehydrated through graded ethanol washes, and stained with hematoxylin and eosin. After drying overnight, tissue morphology was examined under a light microscope.

#### Immunohistochemical (IHC) staining

To assess the impact of 5-ASA@Cm-Gel’:Cm-CNC’ NF on intestinal barrier integrity, IHC analysis was performed to evaluate the expression of tight junction proteins, including Occludin, Claudin-5, and ZO-1 [6, 8, 60]. Rehydrated sections underwent antigen retrieval and were incubated overnight at 4 °C with primary antibodies specific to each target protein. Occludin and Claudin-5 were visualized using the HRP/DAB ABC detection kit (Abcam), while ZO-1 was detected using an HRP-conjugated secondary antibody and DAB substrate with nickel enhancement (Vector Labs). Colon tissues were stained with Alcian blue (pH 2.5) to visualize acidic mucins and assess mucus coverage. Briefly, samples were fixed, stained for 30 min at room temperature, washed to remove excess dye, and imaged microscopically [60]. All slides were counterstained with hematoxylin, dehydrated in graded ethanol, cleared with Histo-clear, and mounted with DPX mounting medium. Digital images were captured using a Carl Zeiss digital slide scanner.

### Statistical analysis

All data were expressed as mean ± standard deviation (SD). Statistical comparisons were performed using independent experiments. All data are expressed as mean ± standard deviation (SD) and analyzed with GraphPad Prism 9.5. Differences between groups were compared via one-way ANOVAs or Student’s t-test. ∗*P* < 0.05, ∗∗*P* < 0.01, ∗∗∗*P* < 0.001.

## Results and discussion

### Synthesis of Cm-Gel and Cm-CNC

#### Characterization of Coumaroyl-Modified Gelatin (Cm-Gel)

The synthesis of Cm-Gel was carried out via a carbodiimide-mediated coupling reaction, where gelatin was modified with coumaric acid. The successful conjugation was first confirmed through ¹H-NMR analysis (Fig. S1). In the spectrum of unmodified gelatin, characteristic peaks were observed at 3.2 ppm corresponding to the amino residue protons of lysine. In comparison, methyl protons of amino acids appeared in the range of 0.8–1.2 ppm. The aromatic protons of phenylalanine residues were detected between 7.3 and 7.5 ppm. Upon modification with coumaric acid, the Cm-Gel spectrum exhibited significant changes. In addition to the original gelatin peaks, new signals were observed in the 7.2–7.8 ppm range, corresponding to the aromatic protons of the coumaroyl group. The presence of both gelatin-specific and coumaroyl-specific peaks indicated the successful formation of an amide bond between gelatin and coumaric acid.

Further confirmation was obtained from FT-IR spectroscopy (Fig. S3a). In the Cm-Gel spectrum, a broad band at 3300 cm⁻¹ was attributed to O–H and N–H stretching, suggesting the coexistence of phenolic and amide functionalities. The C–H stretching vibrations were observed at 2940 cm⁻¹. A prominent peak at 1640 cm⁻¹, corresponding to amide I (C = O) stretching, was more intense in Cm-Gel, further confirming amide bond formation. Additionally, peaks at 1449 cm⁻¹ and 1235 cm⁻¹ were assigned to C–O stretching and C–N stretching, respectively. A series of bands between 900 and 700 cm⁻¹ indicated C–H out-of-plane bending typical of aromatic moieties, confirming the incorporation of the coumaric acid group. The degree of coumaric acid (Cm) conjugation onto gelatin (Cm-Gel) was quantified by UV-Vis spectroscopy at 280 nm using a standard calibration curve. The Cm content was approximately 4.8% w/w, confirming successful grafting via amide bond formation.

#### Characterization of coumaroyl-modified Cellulose Nanocrystals (Cm-CNC)

**Fig. 1:**
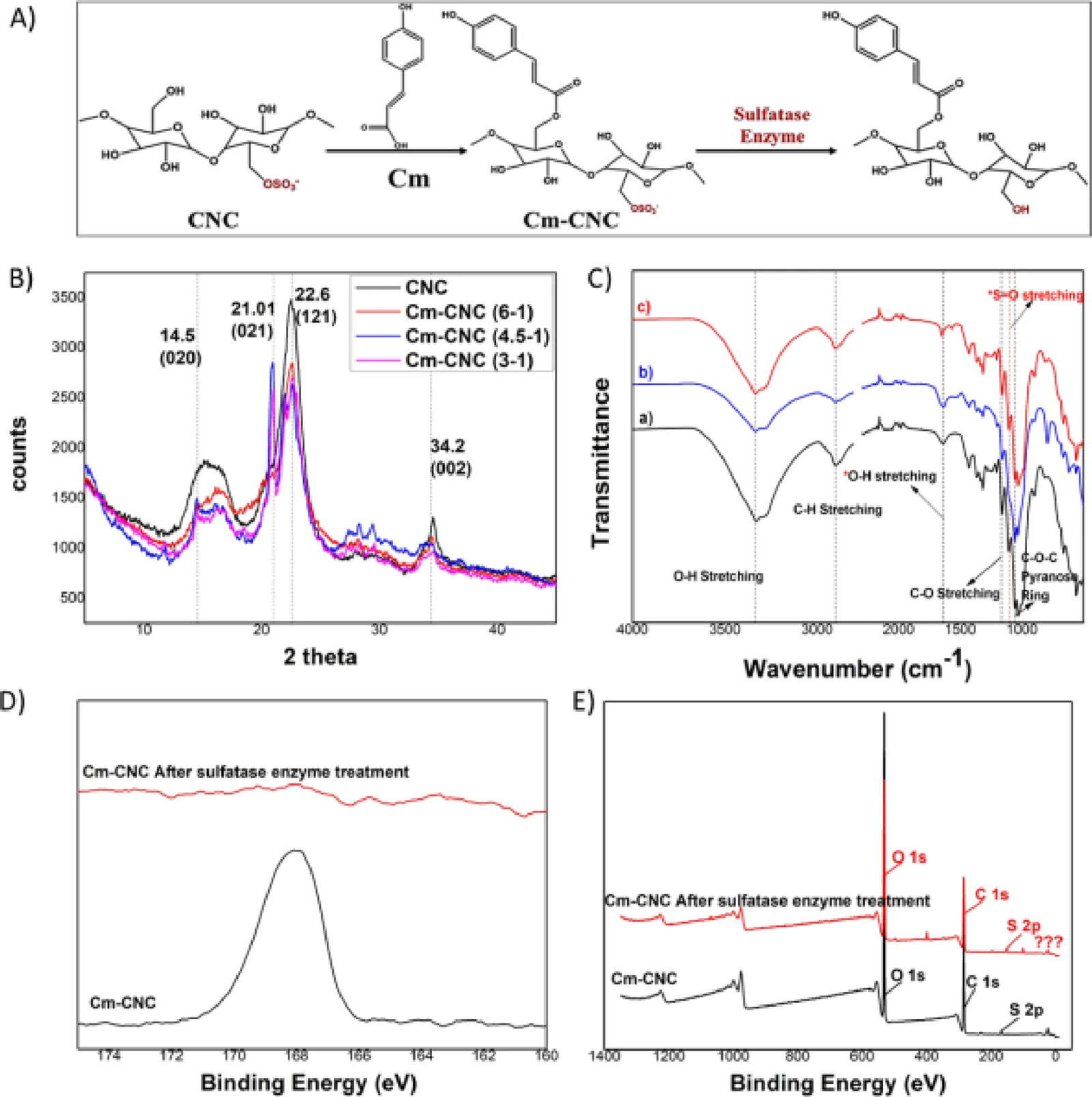
Modification of Cm-CNC and its sulfatase-responsive behaviour. **A**) Schematic representation of the CNC conjugation and sulfatase enzyme response of Cm-CNC. **B**) XRD spectrum of CNC and Cm-CNC prepared. **C**) FT-IR spectrum of (a) CNC, (b) Cm-CNC, and (c) sulfatase enzyme-treated Cm-CNC. **D**&**E**) XPS analysis of Cm-CNC and Sulfatase enzyme-treated Cm-CNC for the confirmation of sulphate group removal

The Cm-CNC conjugates were synthesized via esterification reaction between coumaric acid and cellulose nanocrystals (Fig. 1A). The conjugation was performed using varying molar ratios of coumaric acid to CNC, such as 3 − 1, 4.5-1, and 6 − 1, and confirmed by ¹H-NMR (Fig. S2). The ¹H-NMR spectrum of unmodified CNC displayed characteristic glucose proton signals at 3.03, 3.54, 3.76, and 4.63 ppm, corresponding to H4, H2, H3/5/6, and H1 of the anhydroglucose units [61]. Additional peaks at 4.29, 5.36, and 5.41 ppm were attributed to residual hydroxyl protons at positions 6, 3, and 2, respectively (Fig. S2B). After conjugation, all Cm-CNC variants showed new peaks in the aromatic region (7.0–8.0 ppm), confirming the presence of coumaroyl groups. Specific peaks at 6.36 ppm and 7.52 ppm were attributed to = CH–CO– and –C = CH– protons, respectively, of the coumaroyl moiety (Fig. S2C). The glucose proton signals remained mostly unchanged, though slight reductions in intensity of the 2-H, 3-H, and 6-H signals suggested the covalent attachment of coumaroyl groups to hydroxyl sites on the CNC backbone.

The FT-IR spectra provided further evidence of successful conjugation. For CNC, strong O–H stretching bands appeared at 3331, 3396, and 3286 cm⁻¹, which were also present in Cm-CNC samples (Fig.S3B). New peaks at 3012 cm⁻¹ and 2805 cm⁻¹ in Cm-CNCs were assigned to aromatic C–H stretching from the coumaroyl groups. Notably, the 3012 cm⁻¹ peak was absent in unmodified CNC, strongly supporting successful modification. A distinct ester carbonyl stretching peak appeared at 1644 cm⁻¹ in Cm-CNC. Though ester C = O typically appears above 1680 cm⁻¹, this downshift is attributed to hydrogen bonding interactions within the cellulose structure, which increases the polarity of the ester carbonyl bond, causing a shift to lower wavenumbers [62]. Such a shift is well-documented and serves as further evidence for the successful esterification of CNC with coumaric acid.

X-ray diffraction (XRD) analysis was conducted to evaluate the crystalline structure of CNC before and after modification with coumaric acid. Figure 1B shows the XRD patterns for unmodified CNC and Cm-CNCs synthesized at Cm-CNC molar ratios of 6 − 1, 4.5-1, and 3 − 1. The unmodified CNC sample exhibited characteristic diffraction peaks at approximately 2θ = 14.5°, 21.0°, 22.6°, and 34.2°, which are indexed to the (020), (021), (121), and (002) crystallographic planes of cellulose Iβ, consistent with previously reported data for highly crystalline cellulose. The sharp and intense peak at 22.6° (121) reflects the predominant crystalline domain, confirming the semi-crystalline nature of CNCs. Upon chemical modification with coumaric acid, the overall intensity of these diffraction peaks decreased in all Cm-CNC samples, indicating a reduction in crystallinity. This decline suggests successful surface functionalization via esterification, which disrupts the hydrogen bonding network and partially disturbs the ordered crystalline regions [62, 63]. Among the Cm-CNC samples, the effect was more pronounced as the coumaric acid content increased, particularly in the Cm-CNC (3 − 1) formulation, where the diffraction peaks became noticeably broader and less intense. Despite this reduction, the preservation of key diffraction positions in all modified samples suggests that the core crystalline structure of cellulose Iβ was largely retained. The absence of any new crystalline peaks further supports that the coumaroyl modification occurred primarily on the amorphous or surface regions of the CNCs, rather than inducing a complete structural transformation [63]. These results confirm that coumaric acid was successfully grafted onto the CNC surface, and the extent of modification was correlated with a modest reduction in crystallinity. This partial amorphization may be beneficial for improving matrix compatibility, facilitating drug loading, and enhancing the responsiveness of the Cm-CNCs in the final nanofiber system. Cm content in esterified Cm-CNC derivatives was quantified using UV-Vis spectroscopy in DMSO at 280 nm, based on a separate Cm standard curve. The Cm content was 6.5% for Cm-CNC (3 − 1), 15.2% for (4.5-1), and 13.2% for (6 − 1), with Cm-CNC (4.5-1) showing the highest conjugation efficiency. This formulation was selected for all subsequent experiments and is referred to as Cm-CNC throughout the manuscript. The analysis confirmed successful coupling and enabled comparative evaluation of modification efficiency across formulations.

The degree of photodimerization of coumaroyl moieties was assessed for Cm-Gel and Cm-CNC under UV irradiation (254 nm, 6 W). As shown in figure S1C, Cm-Gel displayed a rapid increase in dimerization, reaching 10.8% within 20 min and plateauing at 13.5% by 70 min, while Cm-CNC (3:1) showed a much lower conversion of 4.5%. The higher dimerization efficiency of Cm-Gel is likely due to the greater molecular flexibility and accessibility of coumaroyl residues in the gelatin matrix, facilitating [2 + 2] cycloaddition. In contrast, the rigid crystalline structure of CNCs may hinder optimal alignment of coumaroyl groups, resulting in reduced dimer formation. These results suggest that Cm-Gel offers superior UV-crosslinking potential, while Cm-CNC contributes moderately to photo-responsiveness. Therefore, the complex formed from Cm-Gel and Cm-CNC can undergo UV-induced photo-crosslinking, driven by the inherent dimerization capability of the coumaroyl groups on both components.

### Sulfatase-responsive behaviour of Cm-CNCs confirmed by FTIR and XPS analysis

To confirm the sulfatase enzyme responsiveness of Cm-CNCs, the enzymatic cleavage of sulfate groups was investigated by treating Cm-CNCs with sulfatase at a concentration of 20 U/mL. The resulting structural and compositional changes were analyzed via Fourier-transform infrared (FTIR) spectroscopy and X-ray photoelectron spectroscopy (XPS). The FTIR spectra (Fig. 1C) revealed distinct differences between untreated CNC, Cm-CNC, and Cm-CNC treated with sulfatase. In the spectrum of untreated CNC and Cm-CNC a characteristic sulfate ester peak was observed at ~ 1240 cm¹, corresponding to S = O stretching vibrations. This peak disappeared after enzymatic treatment, indicating successful removal of the sulfate group. Additionally, the relative intensity of C–O and O–H stretching bands was altered, further supporting changes in the surface chemistry due to sulfatase-mediated cleavage. The retention of other cellulose backbone-related peaks, including those associated with C–H vibrations and pyranose ring structures, confirmed that the core cellulose structure remained intact after enzymatic treatment. XPS analysis provided further evidence of sulfate removal (Fig. 1D&E). The high-resolution scan of the S 2p region (Fig. 1D) showed a prominent peak at ~ 168 eV in Cm-CNC, indicative of the presence of sulfate groups. After sulfatase treatment, this peak was completely absent, demonstrating efficient enzymatic cleavage of the sulfate ester moieties. These results confirm that Cm-CNC possess sulfatase-responsive characteristics, making them an effective platform for site-specific drug delivery to the colon, where sulfatase enzymes are overexpressed due to inflammation like ulcerative colitis. The ability to enzymatically cleave sulfate groups without disrupting the cellulose backbone enables the controlled release of encapsulated drugs or therapeutic agents in response to disease-specific enzymatic cues.

### Complex formation of Cm-Gel with Cm-CNC at varying mass ratios

To evaluate the electrostatic complexation between coumaroyl-modified gelatin (Cm-Gel) and coumaroyl-modified cellulose nanocrystals (Cm-CNC), various mass ratios of Cm-Gel: Cm-CNC were prepared in a solvent system of acetic acid: H_2_O (9:1, v/v). This specific solvent composition was chosen over low-pH deionized water alone, as it reduced the solution viscosity, thereby facilitating the electrospinning process while maintaining the desired acidic environment essential for stable complex formation.

**Fig. 2:**
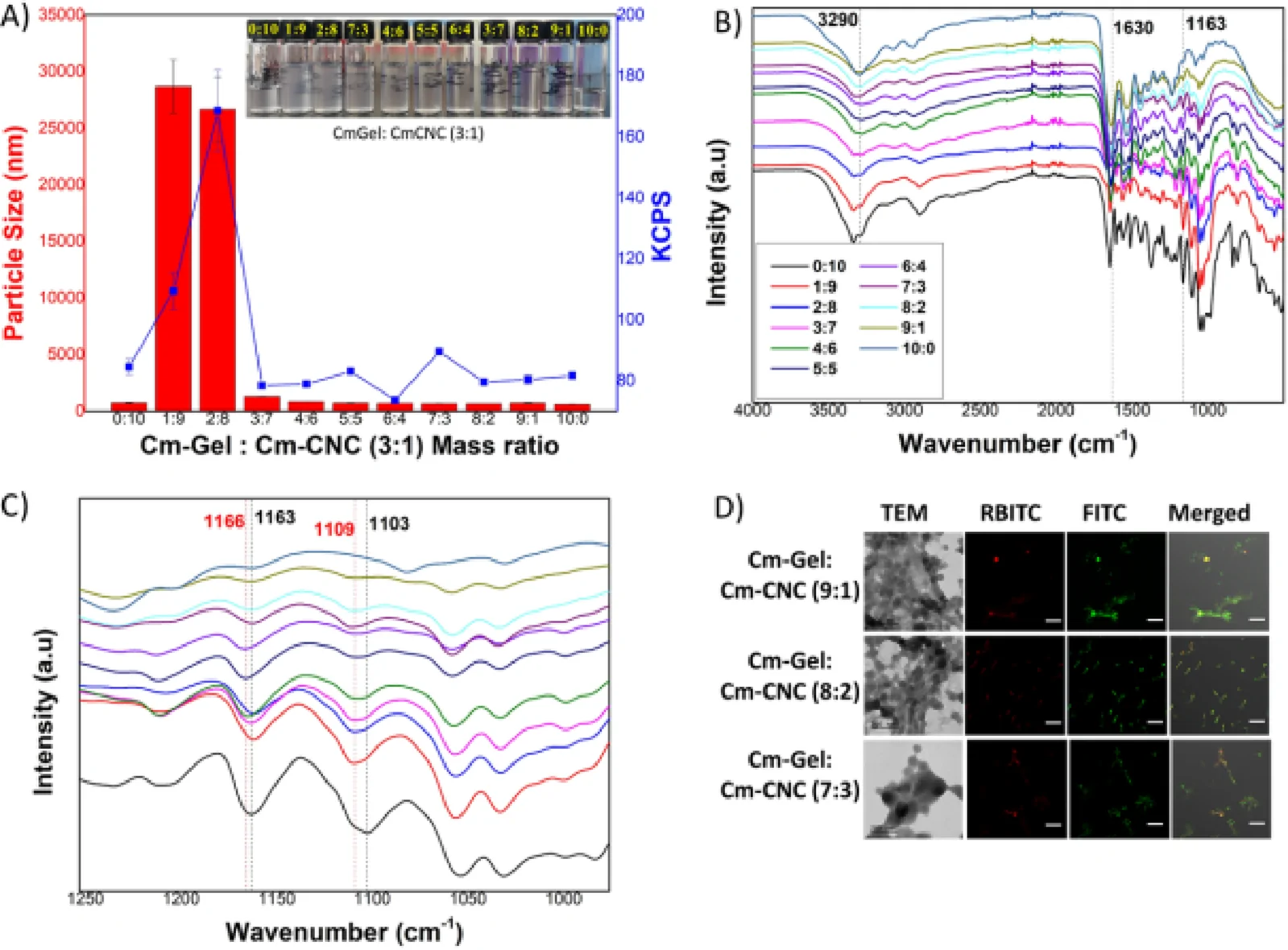
Characterization of the Complex prepared using Cm-Ge and Cm-CNC. **A**) Particle size and light scattering intensity of the complex formation between Cm-Gel and Cm-CNC. **B**) FT-IR spectrum of the complex formed between Cm-Gel: Cm-CNC (x: y) different ratios. **C**) Expanded section of the FT-IR analysis showing the shift in the peak due to the electrostatic interaction. **D**) TEM and Confocal images of the complex formed between Cm-Gel labelled with RBITC and Cm-CNC labelled with FITC in different mass ratio (Scale bar- 50 μm)

Dynamic light scattering (DLS) and light scattering intensity measurements (Fig. 2A) revealed that increasing the Cm-CNC content resulted in a significant increase in particle size, indicating enhanced aggregation and network formation due to stronger electrostatic binding. The maximum particle size was observed at a Cm-Gel: Cm-CNC ratio of 1:1, beyond which the size plateaued or slightly decreased, suggesting saturation of available binding sites or stabilization of complex architecture. FT-IR spectral analysis of the freeze-dried complexes (Fig. 2B&C) confirmed successful electrostatic interactions between the negatively charged sulphate groups of CNC and the positively charged residues of Cm-Gel. Notably, shifts in the S = O stretching peak (~ 1220–1240 cm⁻¹) in the spectra of the complexes compared to the pure Cm-CNC suggest strong ionic interactions, supporting the formation of a polyelectrolyte complex.

Transmission electron microscopy (TEM) images (Fig. 2D) showed well-defined nanoparticulate structures in the complexes, providing visual confirmation of successful self-assembly between Cm-Gel and Cm-CNC. These images demonstrated a transition from loosely aggregated forms in Cm-Gel to more condensed and structured networks upon increasing Cm-CNC content. To further confirm complex co-localization, fluorescently labeled conjugates were employed: Cm-Gel was labeled with Rhodamine B isothiocyanate (RBITC) and Cm-CNC with Fluorescein isothiocyanate (FITC) (Fig. 2D). The merged confocal microscopy images showed distinct yellow to orange fluorescence due to the overlap of red and green channels, indicating spatial colocalization of Cm-Gel and Cm-CNC in the complex. This fluorescence overlay provided strong evidence that both components were incorporated into the same supramolecular structure. These combined results demonstrate that the Cm-Gel and Cm-CNC self-assemble through electrostatic interactions into stable complexes in an acetic acid-rich aqueous environment. This complexation strategy not only confirms the compatibility of the components but also validates the suitability of this system for electrospinning applications aimed at biomedical and drug delivery use.

### Fabrication and morphological characterization of Cm-Gel/Cm-CNC nanofibers

**Fig. 3:**
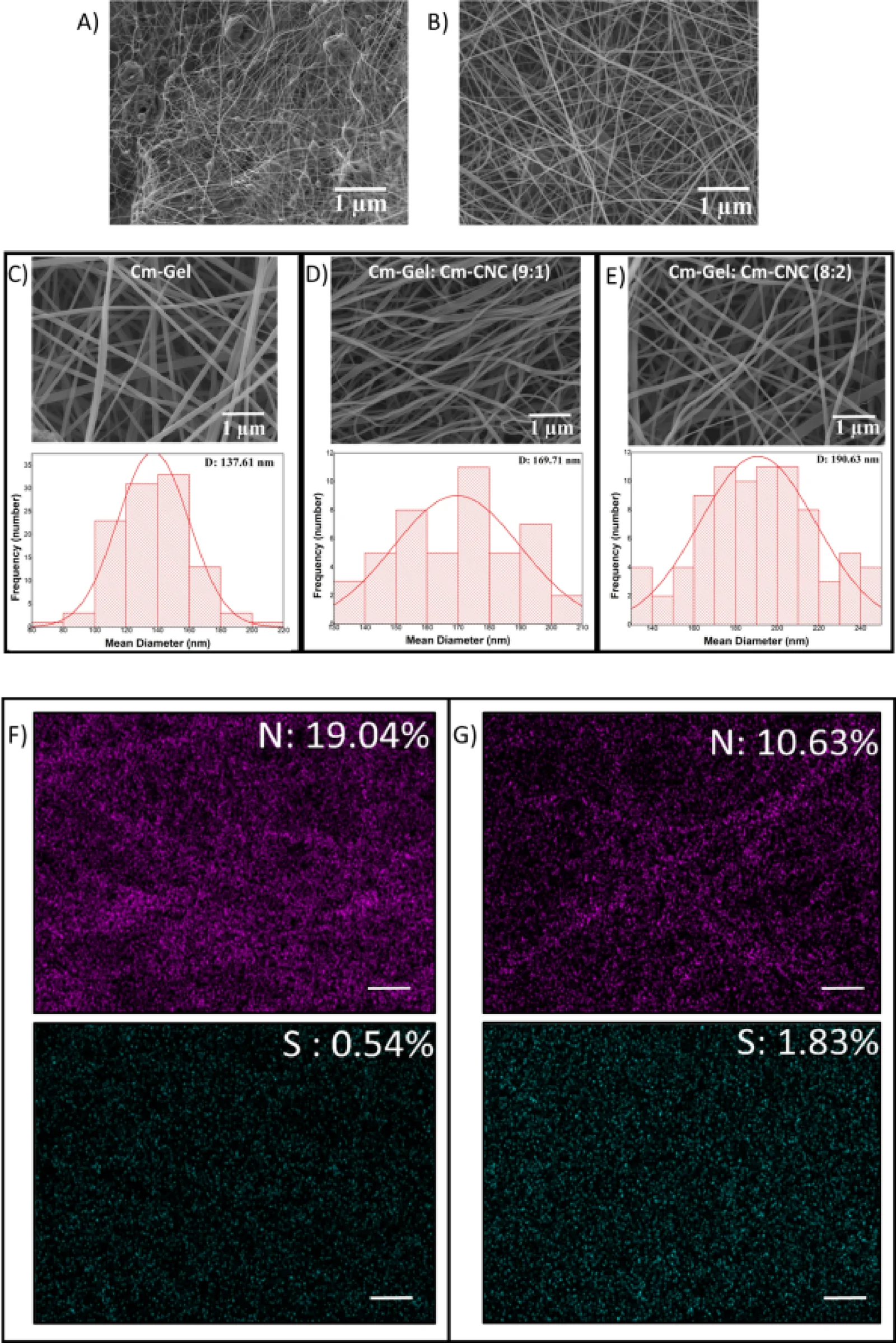
Morphological studies of nanofibers prepared. **A**) SEM image of Cm-Gel-based nanofiber prepared using acetic acid: H_2_O (9:1) solvent, **B**) SEM image of Cm-Gel nanofiber prepared using acetic acid: H_2_O (1:1) solvent. **C**) SEM image and diameter analysis of the Cm-Gel nanofiber, **D**) SEM and diameter analysis graph of Cm-Gel: Cm-CNC (9:1) mass ratio. **E**) SEM and diameter analysis of Cm-Gel: Cm-CNC (8:2) mass ratio. **F**&**G**) Elemental analysis of Nitrogen and Sulfur atom percentage from Cm-Gel: Cm-CNC (9:1) Nanofiber and Cm-Gel: Cm-CNC (8:2) nanofiber, respectively (scale – 5 μm)

Electrospun nanofibers were successfully fabricated using Cm-Gel as the primary polymer matrix, with or without the incorporation of Cm-CNC. The electrospinning parameters were optimized using a solvent mixture of acetic acid and deionized water (9:1, v/v), which was critical to achieving stable jet formation and uniform nanofiber morphology (Fig. 3A). In contrast, when a 1:1 acetic acid: H_2_O solvent ratio was employed, the resulting solution displayed increased surface tension and reduced volatility, leading to the formation of droplet-like or beaded structures rather than continuous fibers (Fig. 3B). This observation underscores the importance of solvent composition in controlling viscosity, conductivity, and surface tension to ensure successful fiber formation [64].

Nanofibers made from 20% (w/v) Cm-Gel alone yielded smooth, continuous structures with an average diameter in the range of 200–250 nm (Fig. 3C). To enhance the structural and functional properties of the nanofibers, Cm-CNC was incorporated at concentrations of 1%, 2%, and 3% (w/v), corresponding to Cm-Gel: Cm-CNC mass ratios of 9:1, 8.6:1.4, and 8:2, respectively. Attempts to increase Cm-CNC beyond 3% (w/v) were unsuccessful due to poor dispersion stability, which resulted in particle aggregation and defective electrospinning. Thus, 3% (w/v) was determined to be the maximum dispersible concentration of Cm-CNC in the electrospinning formulation.

**Fig. 4:**
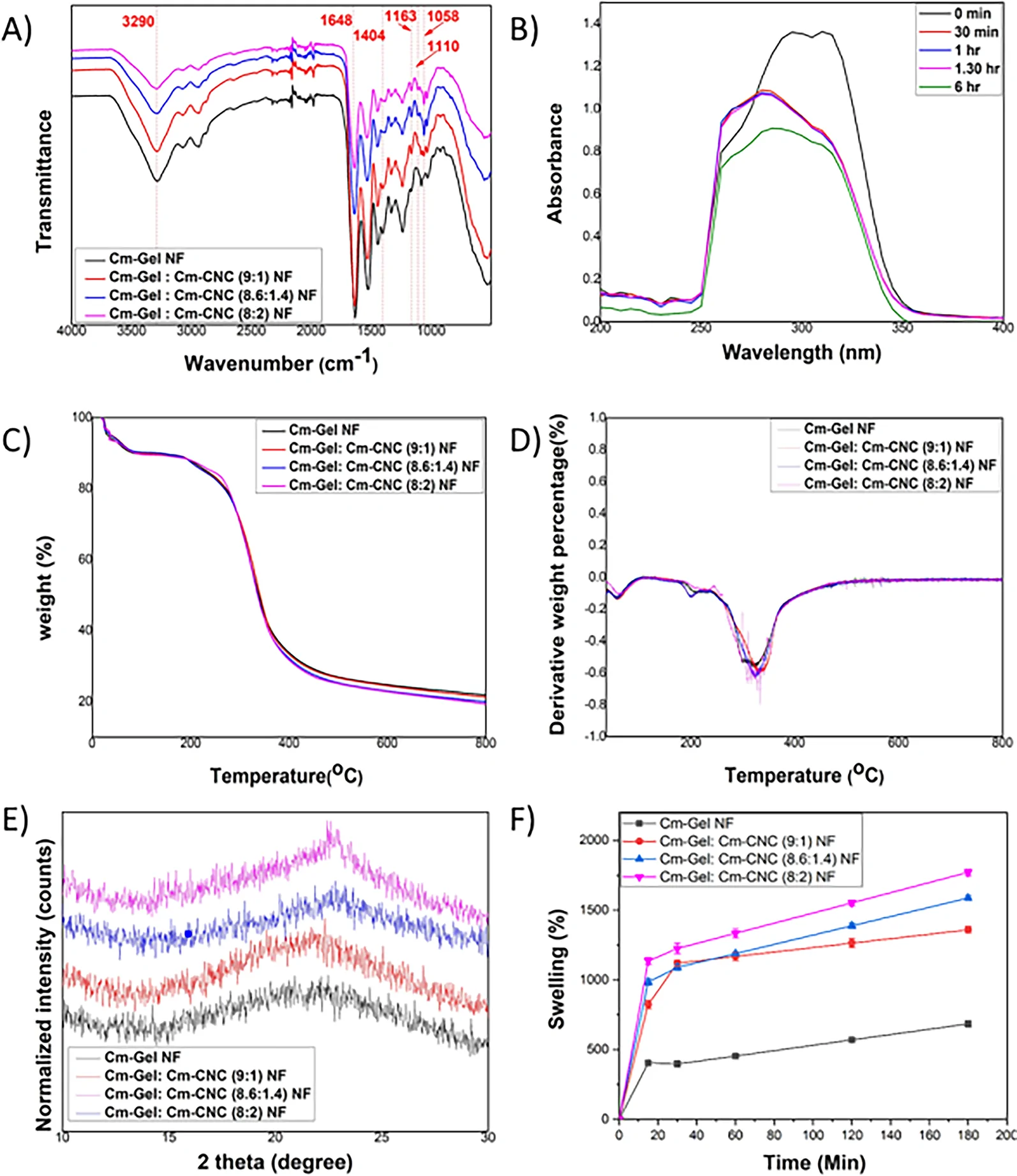

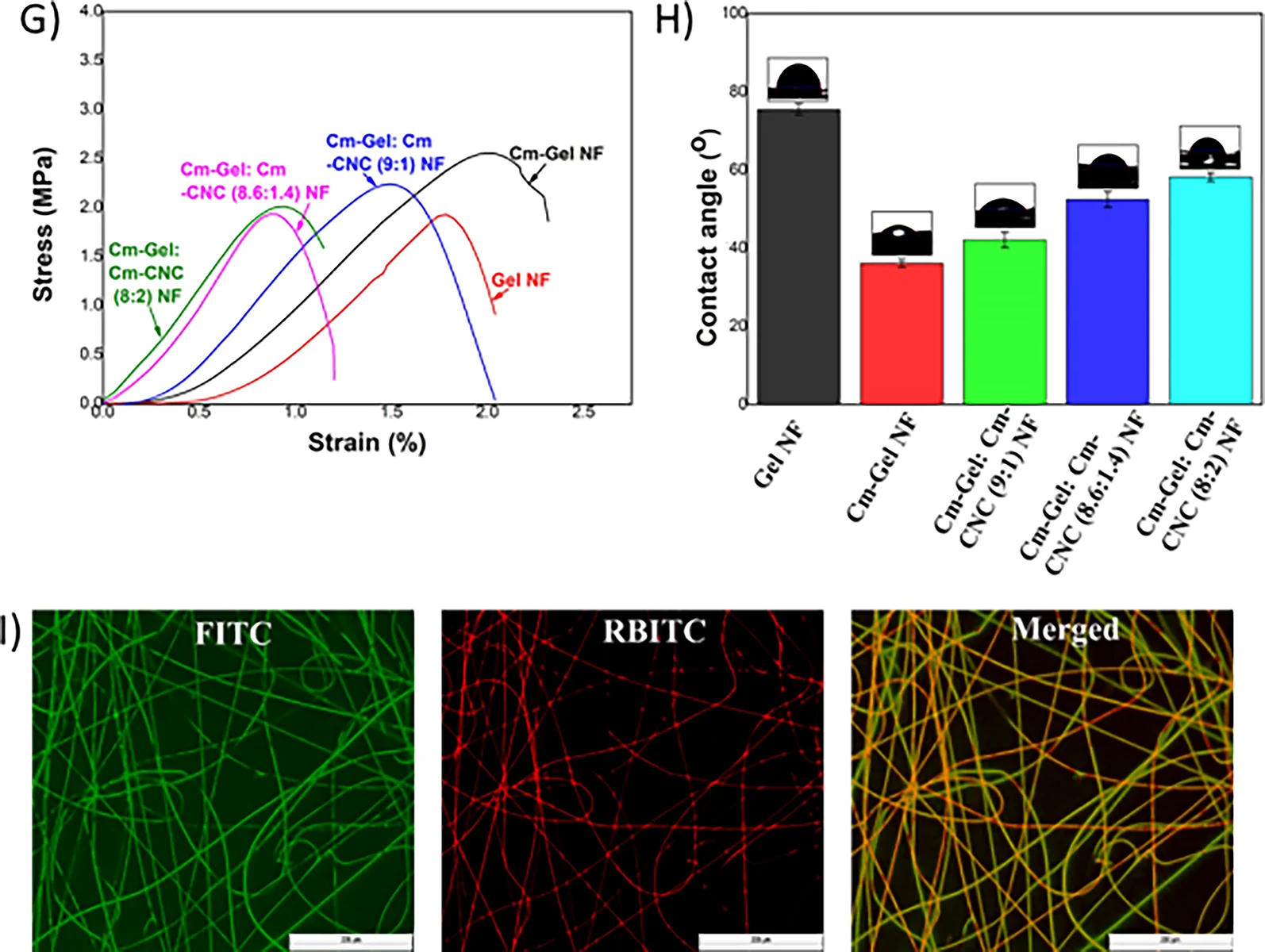
Physico-chemical property analysis of the nanofibers prepared. **A**) FT-IR spectrum of Cm-Gel NF and Cm-Gel: Cm-CNC NF(x: y) ratios, **B**) UV dimerization of the Cm-Gel: Cm-CNC(8:2) NF using UV spectrum, **C**)&**D**) Thermal analysis of the nanofibers prepared of Cm-Gel: Cm-CNC (x: y) NF, **E**) XRD analysis of the nanofibers showing the Cm-CNC incorporation showed slight crystallinity, **F**) Swelling percentage of the nanofibers Containing Cm-Gel NF and Cm-Gel: Cm-CNC NF(x: y) ratios, **G**) Mechanical strength of the nanofibers prepared using various concentration of the Cm-CNC, **H**) contact angle measured for the nanofibers prepared showing the hydrophilicity property of the fibers, **I**) Confocal image of nanofiber prepared using FITC labeled Cm-Gel and RBITC labeled Cm-CNC with mass ratio of Cm-Gel: Cm-CNC (8:2)NF

**Fig. 5:**
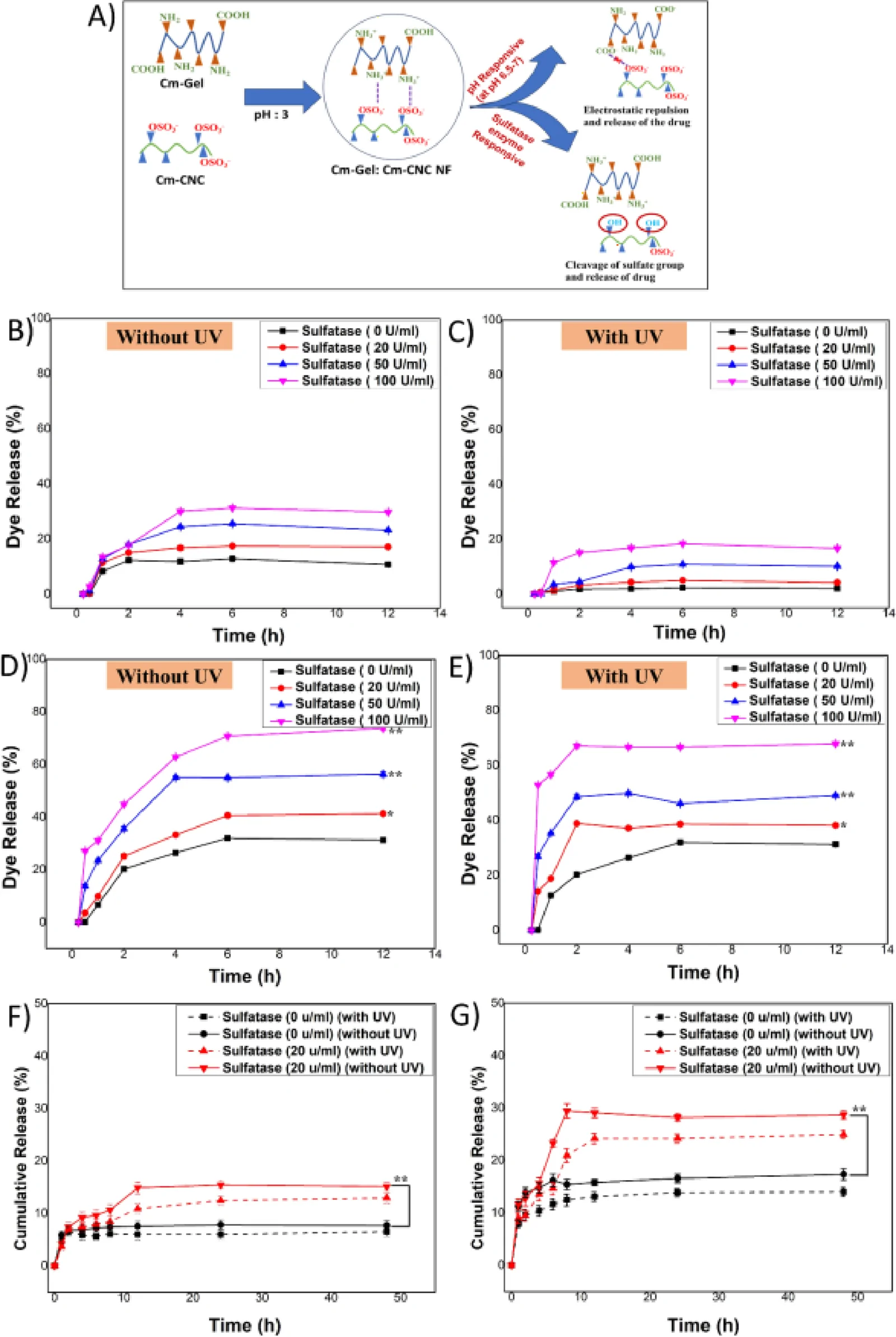
Release profile of Calcein dye with various sulfatase enzyme concentration from Cm-Gel’: Cm-CNC’ NF in **A**) Schematic representation of Cm-Gel: Cm-CNC NF release mechanism in presence of sulfatase enzyme and pH stimulus, **B**) pH 2.1 without UV cross-linking, **C**) pH 2.1 with UV cross-linking, **D**) pH 7 without UV cross-linking, **E**) pH 7 with UV cross-linking, (**F**) Release profile of 5-ASA loaded nanofiber at pH 2.5 using dialysis method with various sulfatase enzymes concentration at pH 2.1, and (**G**) Release profile of 5-ASA@ Cm-Gel’: Cm-CNC’ NF at pH 7 using dialysis varying sulfatase enzymes, (*n* = 3). Significance is denoted as *p < 0.05, **p < 0.005, and ***p < 0.001

**Fig. 6:**
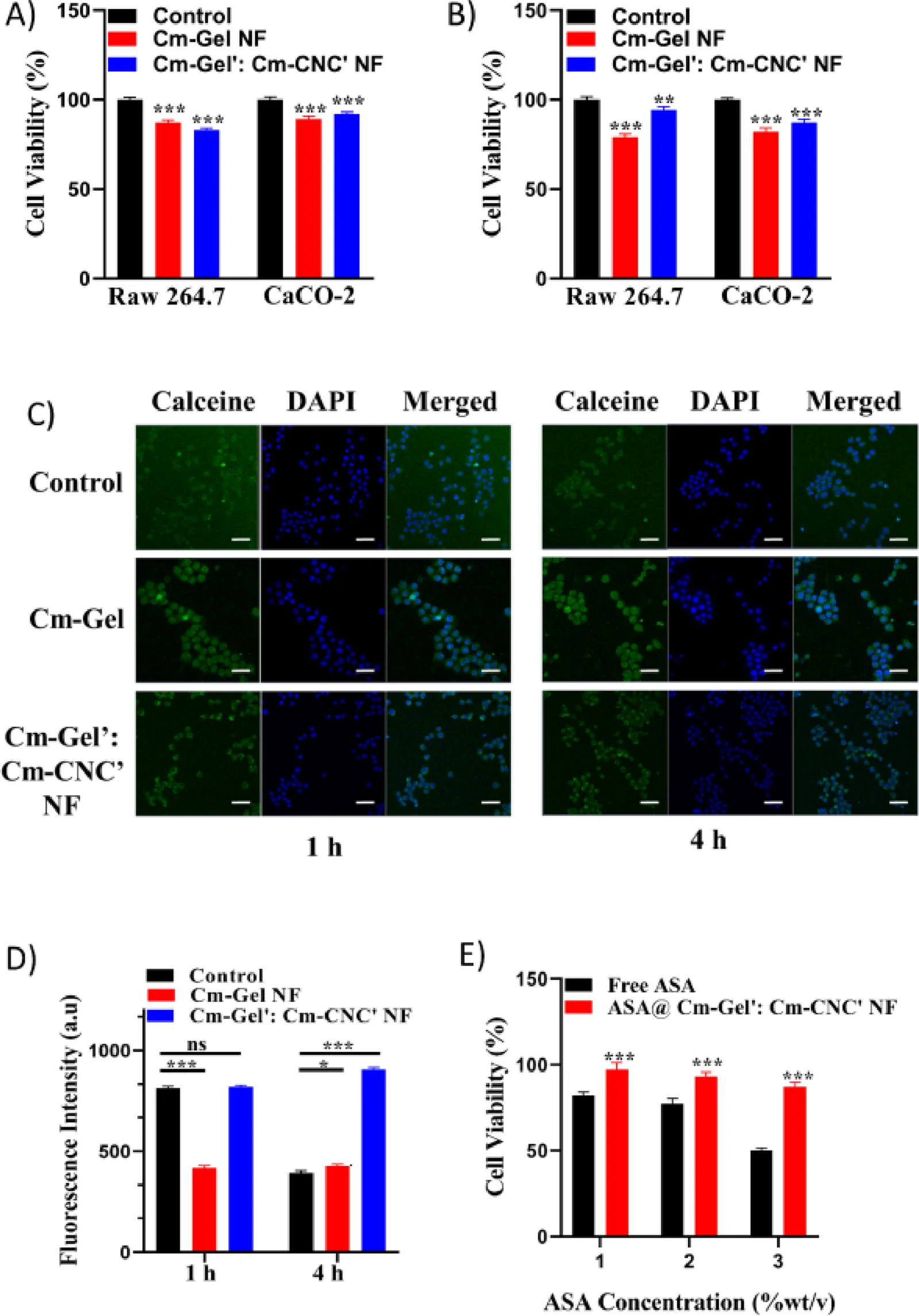
Cellular interaction studies of the nanofibers with Raw 264.7 and CaCO-2 cells. **A**) Cell viability test for the Cm-Gel NF and Cm-Gel’: Cm-CNC’ NF using the direct method, **B**) Cell viability test for the nanofibers using the extract method. **C**) cellular interaction study using a confocal microscope for the nanofiber and Raw264.7 cells (Scale bar − 20 μm). **D**) Summary of Calcein fluorescence intensity in Raw 264.7 cells treated with nanofibers. **E**) Cell viability test of Free ASA and 5-ASA@Cm-Gel’: Cm-CNC’ NF. Data are presented as mean ± SD (*n* = 3). Statistical analysis was performed using one-way analysis of variance (ANOVA) followed by multiple comparison testing in GraphPad Prism to evaluate differences among groups. Significance is denoted as *p < 0.05, **p < 0.005, and ***p < 0.001, while ns indicates no significant difference

**Fig. 7:**
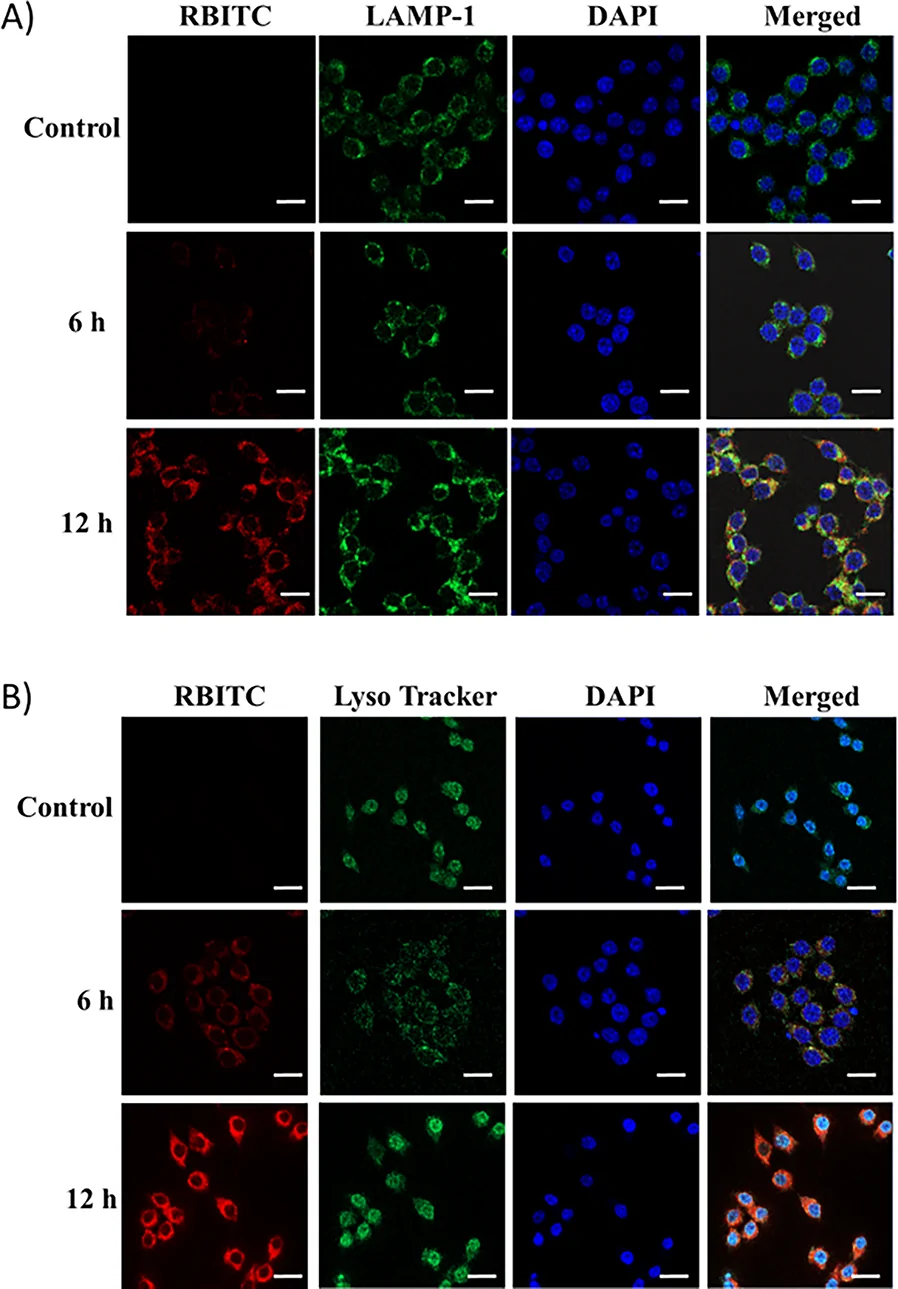

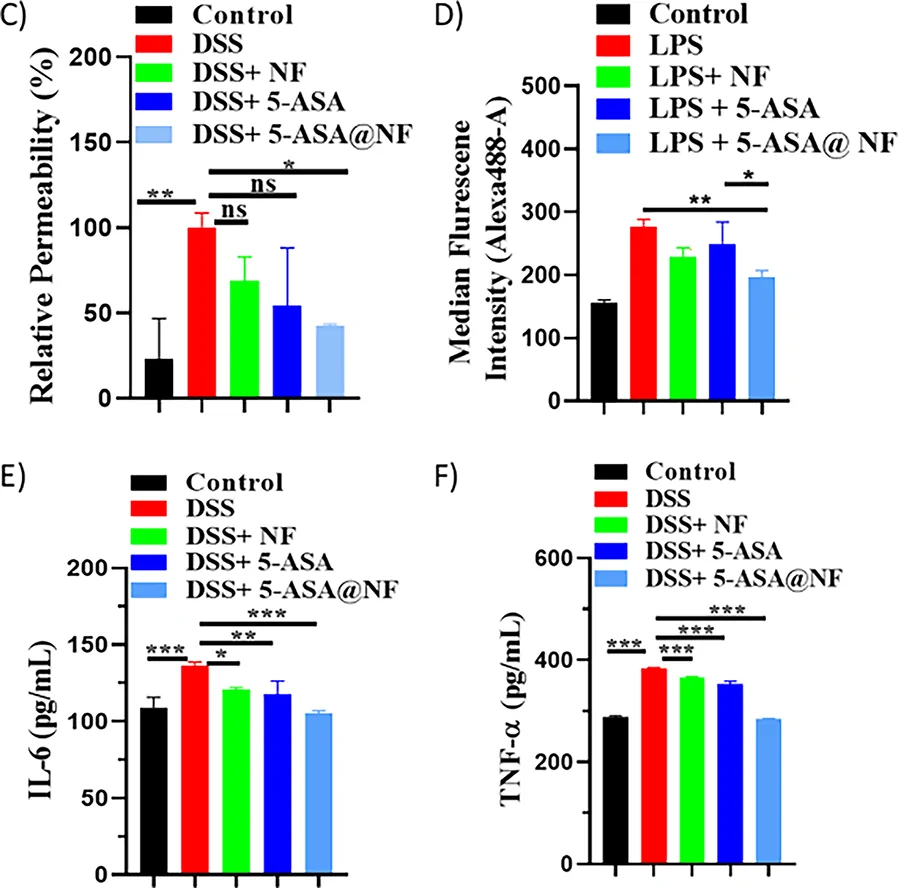
Intracellular trafficking, epithelial barrier restoration, oxidative stress reduction, and anti-inflammatory effects of Cm-Gel′: Cm-CNC′ nanofibers. Confocal images in (**A**) show intracellular localization of RBITC-labeled nanofibers with LAMP-1 staining, demonstrating progressive trafficking into late endosomal/lysosomal compartments at 6 and 12 h. (**B**) LysoTracker Green staining illustrates time-dependent lysosomal acidification changes following nanofiber uptake. (Scale bar − 20µm). (**C**) Relative permeability across DSS-disrupted CaCO-2 monolayers after treatment indicates restoration of epithelial barrier integrity. (**D**) Intracellular ROS levels were quantified by DCFH-DA flow cytometry and expressed as median Alexa488-A fluorescence intensity. (**E**) and (**F**) represent quantitative analysis of IL-6 and TNF-α cytokine expression, respectively, in DSS-treated CaCO-2 cells after treatment. (NF represents Cm-Gel’: Cm-CNC’ NF) Data are presented as mean ± SD (*n* = 3). Statistical analysis was performed using GraphPad Prism by one-way ANOVA followed by multiple comparison analysis against the DSS group. Significance is denoted as *p < 0.05, **p < 0.005, and ***p < 0.001; ns indicates no significant difference

**Fig. 8:**
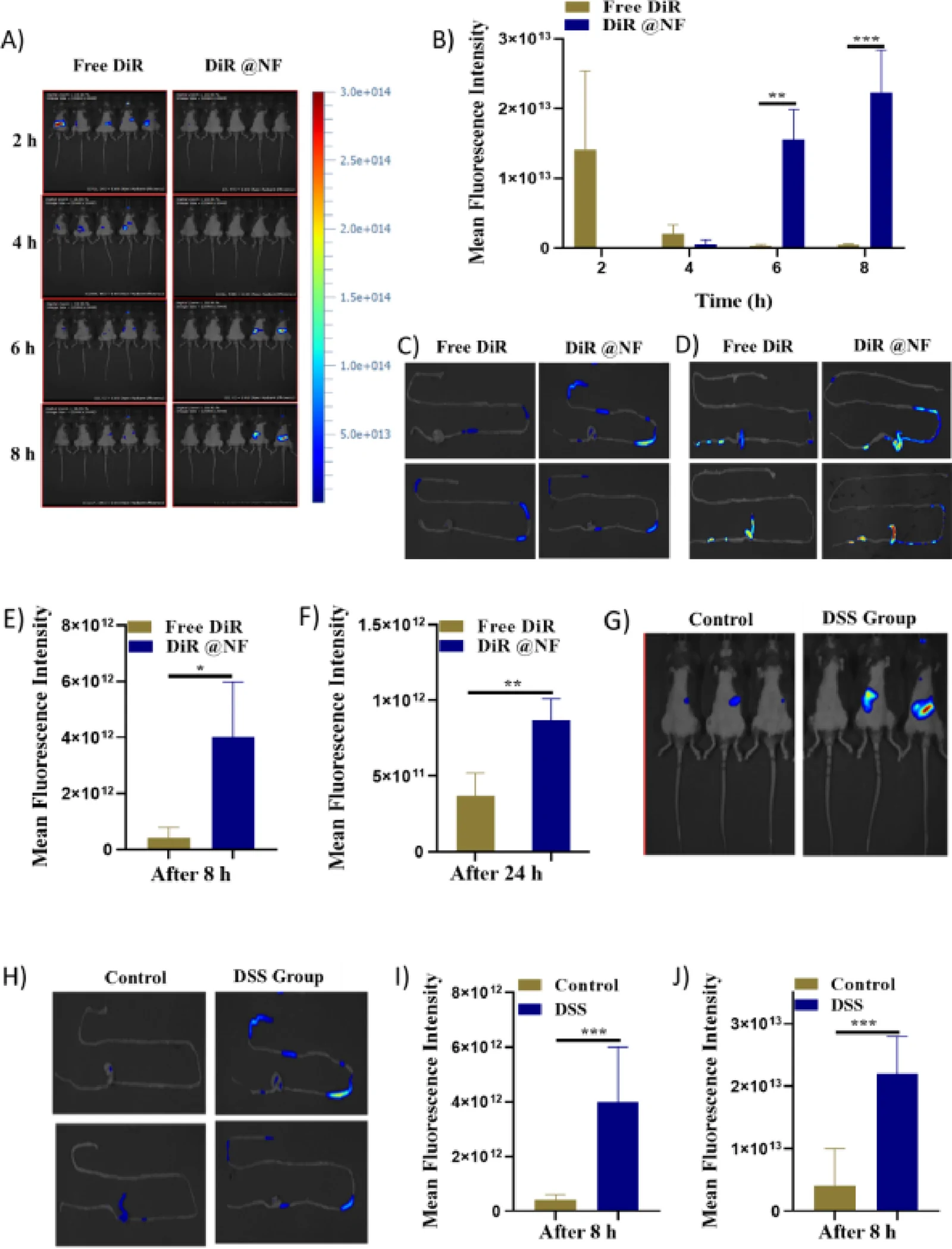
The biodistribution and localisation of the nanofibers in DSS-induced mice. A) Representative images of DiR and DiR@ Cm-Gel’: Cm-CNC’ NF localise in the inflamed GIT region in DSS-treated mice. The DSS-induced mice were orally treated with free DiR and DiR@ Cm-Gel’: Cm-CNC’ NF, and IVIS analysis was performed at different time intervals. **B**) A summary of the fluorescence intensity was plotted by calculating the mean intensity of the mice. Data are presented as Mean ± SD, from a representative experiment (*n* = 5). Two-way ANOVA determined the p-values. *p < 0.05, **p < 0.005, and ***p < 0.001. (**C**-**D**) The mice were euthanized after 8 and 24 h. The ex vivo fluorescence intensity of DiR was checked to assess the biodistribution of the dye and ensure quantifiable signals. (**E**-**F**). A summary of the fluorescence intensity was plotted for 8 and 24 h by calculating the means, Mean ± SD, from a representative experiment (*n* = 5). Student’s t-test determined the p-values. *p < 0.05, **p < 0.005, and ***p < 0.001. (**G**-**H**) Representative images and (**I**-**J**) summary of in vivo and ex vivo analysis of the DiR @ Cm-Gel’: Cm-CNC’ NF in the normal healthy control group and the DSS induced group. (*n* = 5). Mean ± SD, from a representative experiment (*n* = 5). Student’s t-test determined the p-values. *p < 0.05, **p < 0.005, and ***p < 0.001. (NF represents Cm-Gel’: Cm-CNC’ NF)

SEM imaging revealed that the incorporation of Cm-CNC led to a progressive increase in nanofiber diameter compared to Cm-Gel-only fibers, consistent with increased solution viscosity and enhanced electrostatic complexation between the two components. Fiber diameter distributions became broader and shifted toward higher values as the ratio of Cm-Gel: Cm-CNC changed from (9:1) (Fig. 3D) to (8:2) (Fig. 3E) with increasing Cm-CNC content, indicating denser and more entangled fiber networks. These changes are attributed to the electrostatic interactions between the negatively charged sulfate groups on Cm-CNC and the positively charged amino groups of Cm-Gel, which promote intermolecular binding and network formation during fiber stretching [65].

Elemental mapping and energy-dispersive X-ray spectroscopy (EDS) analysis further confirmed the successful incorporation of Cm-CNC into the fiber matrix. Specifically, sulfur (S) signals corresponding to sulfate ester groups on Cm-CNC—increased with higher Cm-CNC content. The sulfur content rose from 0.54 wt% in the 9:1 formulation to 1.83 wt% in the 8:2 formulations (Fig. 3F&G), reflecting a greater density of sulfate-functionalized nanocrystals in the nanofibers. These sulfate groups not only contribute to electrostatic complex formation but also endow the fibers with sulfatase-responsive properties, making them highly suitable for colon-targeted delivery systems. Sulfatase enzymes, which are overexpressed in the inflamed colonic environment of ulcerative colitis, can cleave the sulfate groups, thereby triggering site-specific drug release [66]. Similarly, the intensity of the nitrogen atom decreased from 19.04% to 10.63% enhanced the Cn-CNC content increased and Cm-Gel content decreased. The percentage of oxygen and Carbon atoms also increased as the Cm-CNC content increased (Fig. S4).

In addition to enzymatic responsiveness, the presence of ionizable functional groups in both Cm-Gel and Cm-CNC provides the nanofibers with pH-sensitive behavior, allowing for environmental-triggered swelling and release in the colonic pH range. In contrast, gelatin-only fibers lacked both aromatic coumaroyl and sulfate functionalities, resulting in limited crosslinking capability and absence of stimuli-responsive properties. Therefore, the optimized Cm-Gel: Cm-CNC nanofibers at an 8:2 ratio demonstrated not only improved morphology and mechanical structure but also dual pH- and sulfatase-responsiveness, making them a promising candidate for on-demand drug release applications in inflammatory bowel disease therapy.

### Characterization of Cm-Gel and Cm-CNC nanofibers

FTIR spectra (Fig. 4A) revealed the structural interactions between Cm-Gel and Cm-CNC in the electrospun nanofibers. The Cm-Gel-only nanofiber showed characteristic peaks of amide I and II at ~ 1648 cm⁻¹ and ~ 1540 cm⁻¹, and a broad N–H stretching band at ~ 3290 cm⁻¹ [67, 68]. Upon incorporation of Cm-CNC, the amide I peak exhibited a minor shift, and the N–H stretching band at 3290 cm-¹ showed a decrease in intensity, indicating electrostatic interactions between the gelatin amine groups and the sulfate groups of Cm-CNC. Importantly, a peak at ~ 1110 cm⁻¹ corresponding to the S = O stretching vibration of sulfate esters became more pronounced with increasing Cm-CNC content, confirming the successful incorporation and interaction of Cm-CNC within the gelatin matrix. These spectral changes demonstrate that the increasing concentration of Cm-CNC enhances interfacial bonding and network complexity.

UV-induced photodimerization of the coumaroyl groups was also evident from the UV-Vis absorbance spectra (Fig. 4B), where time-dependent shifts confirmed cis-trans isomerization followed by [2 + 2] cycloaddition. This process created a stable crosslinked network without using chemical crosslinkers, which can compromise biocompatibility. As Cm-CNC content increased, the system retained its photo responsiveness, suggesting compatibility between CNC and the coumaroyl-functionalized matrix. The UV exposure was applied only during the manufacturing stage and not during any in vitro cell experiments, animal studies, or therapeutic administration. Therefore, neither the cells nor the animals were directly exposed to UV irradiation and finalized crosslinked materials were used in subsequent experiments.

Thermogravimetric analysis (TGA) and DTG curves (Fig. 4C & D) demonstrated that all nanofibers underwent a two-step degradation process. The first degradation occurred near 240 °C, likely due to moisture and partial chain scission, and the second major degradation occurred around 360–370 °C due to backbone decomposition. Increasing Cm-CNC content caused minimal variation in degradation onset temperatures, suggesting that the Cm-CNC incorporation did not compromise thermal stability. However, a slight shift toward higher decomposition temperatures with increasing Cm-CNC concentration may be attributed to the reinforcing effect and thermal buffering of crystalline Cm-CNC domains within the gelatin matrix [68].

X-ray diffraction (XRD) patterns (Fig. 4E) showed an amorphous halo centred at ~ 20° in Cm-Gel-only nanofibers. With the addition of Cm-CNC, two distinct peaks appeared at 16.5° and 22.6°, corresponding to the crystalline planes of cellulose Iβ, indicating the preserved crystallinity of CNC. As Cm-CNC content increased, the peak intensity at 22.6° intensified, reflecting an increase in the overall crystallinity of the nanofiber matrix [69]. The unaltered peak positions confirm that CNC retains its native structure during electrospinning. Enhanced crystallinity is associated with improved mechanical strength and dimensional stability.

The tensile stress-strain curves (Fig. 4G) showed a marked improvement in mechanical properties with increasing Cm-CNC content. The Cm-Gel-only fibers displayed lower tensile strength and modulus. The addition of 1% and 2% Cm-CNC increased both tensile strength and stiffness, due to strong interfacial electrostatic interactions and the reinforcing effect of the rigid CNC rods. At 3% Cm-CNC (Cm-Gel: Cm-CNC = 8:2), the fibers exhibited maximum tensile strength, indicating optimal dispersion and binding. However, further increases beyond this may lead to CNC agglomeration and phase separation, causing mechanical properties [67–69]. These trends align with improved structural integrity observed in the morphological and swelling studies.

Water contact angle measurements (Fig. 4H) revealed a decrease from ~ 78° in unmodified Cm-Gel to lower values upon coumaroylation, attributed to the hydroxyl-rich coumaric acid. With increasing Cm-CNC content, the contact angle gradually increased, reflecting reduced surface availability of hydrophilic groups as CNC fills matrix voids and creates a more compact network. The highest Cm-CNC loaded sample (8:2 ratio) exhibited a contact angle close to 65°, suggesting moderate hydrophilicity balanced with water resistance. This balance is beneficial for controlled swelling and sustained release behaviour. Swelling studies (Fig. 4E) showed that Cm-Gel-only fibers swelled rapidly but plateaued early, while CNC-containing fibers absorbed significantly more water over time. The Cm-Gel: Cm-CNC (8:2) nanofibers exhibited the highest swelling ratio, consistent with their increased sulfate content and hydrophilic nature. CNC acts as a sponge within the network, and its hydroxyl groups attract water molecules, enhancing matrix expansion. This increase in swelling correlates directly with the enhanced mechanical properties and surface hydrophilicity up to 3% Cm-CNC [68, 70]. The structural integrity maintained by UV-crosslinking ensures that the fibers retain shape even under high swelling, enabling controlled and localized release of therapeutic agents.

In addition, confocal microscopy was used to visualize the distribution of components within the nanofiber matrix. To confirm nanofiber formation and component colocalization, Cm-Gel and Cm-CNC were labeled with FITC (green) and RBITC (red), respectively (Fig. 4I). The merged images showed clear colocalization, indicating uniform distribution and electrostatic interaction between Cm-Gel and Cm-CNC within the nanofiber matrix.

Furthermore, the dual-drug loading capability of the Cm-Gel: Cm-CNC (8:2) nanofibers was demonstrated using hydrophobic (Nile Red) and hydrophilic (RBITC) fluorescent dyes. Confocal imaging showed successful encapsulation and distribution of both dyes within the nanofibers (Fig. S5A and B). Nile Red-loaded nanofibers showed red fluorescence distributed throughout the structure, confirming compatibility with hydrophobic molecules. Meanwhile, RBITC-loaded nanofibers displayed uniform fluorescence patterns, indicative of hydrophilic dye entrapment. These results support that the nanofiber system is a versatile platform capable of co-encapsulating both hydrophilic and hydrophobic agents, making it a suitable candidate for broad-spectrum oral drug delivery.

The morphology and structural stability of Cm-Gel: Cm-CNC (8:2) nanofibers were evaluated via SEM, both immediately post-fabrication and following long-term storage (Fig. S7A&B). Both freshly prepared and 6-month-stored nanofibers exhibited a continuous fibrous architecture with uniform fiber distribution and no observable structural collapse. While slight surface smoothing and minor fusion between adjacent fibers were observed after long-term storage, the overall network structure remained intact, indicating good morphological stability. This stability is attributable to the crosslinked polymer network and strong intermolecular interactions between Cm-Gel and Cm-CNC, which restrict polymer chain mobility and prevent structural degradation over time [71, 72].

Fig. S7C demonstrates that the mechanical behavior of the Cm-Gel: Cm-CNC (8:2) nanofibers exhibits a significant improvement upon incorporation of Cm-CNC compared to the Cm-Gel only system. The pristine Cm-Gel nanofiber exhibited a gradual increase in stress with increasing strain, indicative of a flexible but mechanically weak structure with relatively low tensile strength. In contrast, the Cm-Gel: Cm-CNC (8:2) nanofibers showed a markedly higher stress at lower strain, suggesting enhanced stiffness and improved load-bearing capacity. This enhancement is attributed to the reinforcing effect of cellulose nanocrystals, which possess high intrinsic mechanical strength and can form strong hydrogen-bonding interactions with the polymer matrix, thereby improving stress transfer efficiency.

To evaluate the behavior of the nanofibers under physiological conditions, they were sequentially exposed to simulated gastric fluid (SGF), simulated intestinal fluid (SIF), and simulated colonic fluid (SCF), each for a duration of 2 h. Following SGF exposure (Fig. S8A), the fibrous structure exhibited swelling and partial fusion, indicative of hydration-induced expansion under acidic conditions. Despite these changes, the overall network integrity was largely maintained, demonstrating resistance to rapid degradation within the gastric environment. This characteristic is critical for the protection of encapsulated drugs during gastric transit.

Upon subsequent exposure to SIF (Fig. S8B), the fibrous morphology became less defined, with smoother surfaces and localized structural collapse observed. These alterations are attributed to increased polymer relaxation and enhanced water uptake at near-neutral pH, resulting in partial disruption of the original fibrous architecture. Comparable pH-dependent swelling and morphological changes have been documented in other hydrophilic polymer systems.

After treatment with SCF (Fig.S8C), the nanofibers displayed pronounced structural loosening, characterized by the development of pores and fragmented regions (highlighted), indicative of progressive degradation and network disintegration under colonic conditions. This response suggests that the nanofiber system is sensitive to the colonic environment, facilitating structural breakdown and promoting drug release at the targeted site. The observed morphological transition from intact fibers to a porous, disrupted matrix supports a controlled release mechanism driven by swelling and matrix erosion.

Taken together, the systematic incorporation of Cm-CNC into Cm-Gel nanofibers results in a robust, photo-crosslinked, and stimuli-responsive platform with tunable swelling, enhanced mechanical strength, and surface properties favorable for colon-targeted drug delivery. Among the tested formulations, the Cm-Gel: Cm-CNC (8:2) ratio exhibited optimal performance across all evaluated parameters, including mechanical integrity, swelling capacity, and structural homogeneity, and was therefore selected for subsequent 5-ASA release experiments, in vitro cell compatibility assays, and in vivo therapeutic efficacy studies. In the further parts of this study, the Cm-Gel: Cm-CNC (8:2) nanofiber formulation will be referred to as Cm-Gel’: Cm-CNC’ NF for clarity and consistency. Throughout the study, two formulations will be compared: Cm-Gel NF (unmodified coumaroyl-gelatin nanofiber) and Cm-Gel’: Cm-CNC’ NF (coumaroyl-gelatin nanofiber reinforced with 3% Cm-CNC).

### Sulfatase and pH-responsive dye release from Cm-Gel’: Cm-CNC’ NF

To evaluate the environmental responsiveness of the nanofibers, calcein-loaded Cm-Gel’: Cm-CNC’ NFs were subjected to dye release tests under different pH conditions (2.1, 4.5, 5.5, and 7.0) and in the presence of varying concentrations of sulfatase enzyme (0, 20, 50, and 100 U/mL), both with and without UV-induced crosslinking. As the colonic alkali pH the sulfate group of the CNC cleaves, disturbing the electrostatic interaction of the Cm-Gel and Cm-CNC. Additional to it, the pH of the colon makes the gelatin with negative charge indicating the dissociation of the complex which enhances the release of the drug (Fig. 5 A). At pH 2.1, representing the acidic gastric environment, both UV-crosslinked and non-crosslinked nanofibers exhibited minimal release due to suppress swelling and low ionization of the matrix. The limited release at this pH can be attributed to the fact that Cm-Gel, having an isoelectric point (pI) around 3.5–3.9, is positively charged at pH below its pI, resulting in tighter interactions with negatively charged CNCs and reduced matrix expansion [73–75]. The non-crosslinked fiber released ~ 10.6% calcein after 6 h (Fig. 5B), while the UV-crosslinked nanofiber showed only 2.2%, confirming the stabilizing effect of photodimerization (Fig. 5 C). Upon enzyme exposure, the release increased in a concentration-dependent manner. At 100 U/mL sulfatase, release rose to 18.3% in crosslinked and 31.2% in non-crosslinked samples. This trend confirms that sulfatase cleaves the sulfate groups from Cm-CNC, disrupting electrostatic interactions and loosening the fiber matrix. However, in UV-crosslinked fibers, photodimerization limited this disintegration, enabling controlled release despite enzyme-mediated degradation, a critical feature for oral administration.

At pH 4.5, which is near the isoelectric point of Cm-Gel, the matrix begins transitioning to a net negative charge. UV-crosslinked nanofibers released ~ 6.7% without sulfatase, while non-crosslinked ones released ~ 13.4%. With increasing sulfatase concentration, the release rose to ~ 32.5% (crosslinked) and ~ 47.8% (non-crosslinked) at 100 U/mL (Fig. S9A and B). These results suggest increasing matrix destabilization as the charge interactions shift, and sulfatase-mediated cleavage becomes more effective. The UV-induced photodimerization, however, continues to restrict over-release, offering a buffered and stable release profile during early intestinal transit.

At pH 5.5, where Cm-Gel is negatively charged and electrostatic repulsion with CNC increases, swelling is further enhanced. The crosslinked fibers released ~ 11.2% without sulfatase and up to ~ 45.3% at 100 U/mL sulfatase. The non-crosslinked samples increased from ~ 19.6% to ~ 58.1% (Fig. S9C&D). The combined effect of negative-negative repulsion and enzymatic cleavage resulted in increased fiber loosening and dye diffusion. Yet, the UV-crosslinked network preserved structural cohesion, validating the importance of crosslinking for sustained release under transitional pH environments.

At pH 7, mimicking colonic conditions, the net negative charge on both Cm-Gel and Cm-CNC caused significant electrostatic repulsion, facilitating high swelling and rapid payload release. UV-crosslinked Cm-Gel’: Cm-CNC’ NFs released ~ 27.9% without sulfatase, while non- crosslinked samples showed ~ 31.2% release. Sulfatase treatment at 100 U/mL further elevated the release to 67.8% (crosslinked) and 73.7% (non-crosslinked) (Fig. 5D & E). This strong release response results from sulfate group cleavage and ionic dissociation, where negatively charged fiber components repel and disrupt the matrix. Importantly, UV crosslinking delays total release, enabling a controlled, on-demand release mechanism ideal for colon-targeted delivery.

Overall, the study highlights the dual stimuli-responsive behavior of Cm-Gel’: Cm-CNC’ NFs driven by both pH and sulfatase. The pH-responsiveness is intrinsically linked to the isoelectric point of Cm-Gel, which governs its surface charge in different pH environments. Simultaneously, the sulfatase enzyme cleaves sulfate groups from Cm-CNC, disrupting electrostatic complexes and promoting payload release. UV-induced photodimerization serves as a crucial mechanism for matrix stabilization, providing a structurally resilient and biocompatible platform for prolonged retention in the colon and therapeutically relevant, site-specific release of loaded agents.

### 5-ASA loading and sulfatase-responsive release behavior

The loading efficiency of 5-aminosalicylic acid (5-ASA) into Cm-Gel′: Cm-CNC′ nanofibers was quantified by UV–Vis spectroscopy. A standard calibration curve constructed at 330 nm showed excellent linearity (R² = 0.9983). Three formulations containing increasing 5-ASA concentrations (0.25, 0.5, and 1.0 wt/v%) were evaluated, yielding encapsulation efficiencies (EE) of 90.52%, 96.07%, and 78.64%, respectively. The highest loading content (LC = 4.10%) was observed at 1.0 wt/v%, whereas the 0.5 wt/v% formulation exhibited the most favorable balance between EE and LC (Table 1). Although increasing the drug concentration to 1.0 wt/v% enhanced total loading capacity, the reduced EE suggests partial drug saturation within the polymer matrix, which may lead to uneven drug distribution and reduced encapsulation stability. Moreover, excessive drug incorporation can adversely affect electrospinning behavior by disturbing polymer chain interactions, resulting in irregular fiber morphology and a higher risk of burst release. Cytocompatibility considerations further supported the selection of the intermediate loading level, as maintaining both formulation stability and biological safety is essential for oral administration. Therefore, the UV-crosslinked Cm-Gel′: Cm-CNC′ nanofiber loaded with 0.5 wt/v% 5-ASA was selected as the optimal formulation, as it provided high encapsulation efficiency, adequate loading capacity, preserved nanofiber integrity, and controlled release suitability for subsequent in vitro, ex vivo, and in vivo studies related to oral colon-targeted delivery.

**Table 1:** Encapsulation efficacy and loading capacity of the nanofiber prepared

Formulation	Cm-Gel (wt/v)	Cm-CNC (wt/v)	5-ASA (wt/v)	EE	LC
Cm-Gel’: Cm-CNC’ NF	20	3	0.25	90.5216	1.14053
	20	3	0.5	96.0772	2.35421
	20	3	1	78.6486	4.098791

To evaluate the release profile of the therapeutic payload, 5-ASA release studies were conducted using a dialysis membrane method in phosphate-buffered saline (PBS, pH 7.4) at 37 °C. Release experiments were carried out at 0 U/mL (control) and 20 U/mL sulfatase enzyme levels. The selected concentration of 20 U/mL reflects physiological relevance, as supported by literature indicating elevated mucin sulfatase activity in the colon during active UC. For instance, median sulfatase activity levels in UC patients have been reported as high as 146 U/g in inflamed tissue, compared to 42.2 U/g in inactive UC and 11.3 U/g in healthy individuals [66]. These values highlight the disease-specific enzyme-rich microenvironment that justifies sulfatase-responsive drug delivery systems. This setup enabled evaluation of basal diffusion and sulfatase-responsive degradation-mediated release.

In the absence of sulfatase at pH 2.1, the UV-crosslinked Cm-Gel’: Cm-CNC’ NF showed a gradual and sustained release profile, attributed to the dense polymeric network formed by UV-induced photodimerization (Fig. 5 F). This crosslinked structure restricted the diffusion of encapsulated 5-ASA, contributing to long-term release over several h. In the presence of 20 U/mL sulfatase at pH 7, a marked enhancement in drug release was observed, indicating effective enzymatic cleavage of sulfate groups from Cm-CNC along with the gelatin and sulfated CNC electrostatic repulsion due to negatively charged (Fig. 5G). This cleavage disrupted the nanofiber network, enhancing matrix loosening and facilitating drug diffusion. Notably, the crosslinked matrix still maintained a degree of structural stability, ensuring controlled, enzyme-triggered release rather than burst release.

The dual behavior sustained release under neutral conditions and accelerated release under sulfatase exposure, demonstrates the therapeutic adaptability of the system. The enzyme-responsiveness, triggered by disease-specific sulfatase expression in UC pathology, ensures targeted drug activation within the colonic lumen, minimizing premature release and systemic exposure. Moreover, the UV-stabilized nanofiber provides prolonged physical retention in the colon, favoring extended therapeutic action. In addition to sulfatase-responsiveness, the nanofiber system also exhibited significant pH-responsiveness, owing to the presence of ionizable groups in Cm-Gel and the CNC matrix. As the pH increases beyond the isoelectric point of Cm-Gel (pH 3.5–4.5), the polymer becomes negatively charged, enhancing electrostatic repulsion with similarly charged Cm-CNC and facilitating fiber swelling. This behavior further promotes the controlled release of 5-ASA under colonic pH conditions (pH ~ 7), complementing the enzyme-triggered mechanism.

### In vitro release study under simulated gastrointestinal conditions and release kinetic modeling

The release profile of 5-ASA from Cm-Gel′: Cm-CNC′ nanofibers was investigated under sequential simulated gastrointestinal conditions to evaluate their colon-targeting potential (Fig. S9E). During exposure to SGF (pH 1.2), drug release was minimal, indicating that the crosslinked polymer network effectively limited premature drug diffusion under acidic conditions, thereby protecting the encapsulated drug during gastric transit. Upon transition to SIF (pH 6.8), a moderate increase in drug release was observed, likely due to hydration and partial swelling of the polymer matrix. Nevertheless, the release remained controlled, suggesting that the nanofiber structure retained sufficient integrity to prevent rapid drug loss in the small intestine. In contrast, a significant acceleration in drug release occurred under SCF (pH 7.4), with cumulative release increasing markedly over time. This effect was further amplified in the presence of sulfatase (50 U/mL), indicating enzyme-responsive degradation or relaxation of the nanofiber matrix. The enhanced drug release in SCF, particularly under enzymatic conditions, can be attributed to increased polymer chain mobility, matrix loosening, and partial network degradation, which facilitate drug diffusion. These findings correlate with SEM observations showing progressive structural disruption and pore formation following sequential exposure to SGF, SIF, and SCF. Collectively, the results demonstrate that the nanofiber system exhibits both pH- and enzyme-responsive behavior, enabling targeted drug release within the colonic environment.

To further clarify the release mechanism, the release data were analyzed using multiple kinetic models, including zero-order, first-order, Higuchi, and Korsmeyer- Peppas models (Table 2). The Korsmeyer- Peppas model yielded the best fit, with correlation coefficients of R² = 0.97 and 0.98 for the conditions without and with 50 U/mL sulfatase, respectively. The calculated release exponent values were *n* = 0.61 (absence of sulfatase) and 0.66 (presence of sulfatase), indicating that drug release follows a non-Fickian (anomalous) transport mechanism governed by a combination of diffusion and polymer relaxation. The slightly elevated n value and release rate constant (k_p_) observed in the presence of sulfatase suggest that enzymatic activity promotes matrix relaxation and enhances drug release under colonic conditions.

**Table 2:** Release kinetic studies od 5-ASA in gastrointestinal simulated conditions

Model	Equation	Parameter (0 U/mL) Sulfatase	*R*² (0 U/mL) sulfatase	Parameter (50 U/mL) sulfatase	*R*² (50 U/mL) sulfatase
Zero-order	Q = K_0_*t*	k_0_ = 2.92	0.74	k_0_ = 3.36	0.77
First-order	LnQ = *Ln*Q_0_-k_1_*t*	k_1_ = 0.18	0.82	k = 0.21	0.86
Higuchi	Q= k_H_t^1/2^	k_H_ = 18.5	0.91	k_H_ = 20.7	0.94
Korsmeyer-Peppas	LnQ = nLnt + Lnk_*p*_	k_p_ = 0.58 *n*=0.61	0.97	k_p_ = 0.62 *n*=0.66	0.98

Although the Higuchi model also demonstrated reasonably high correlation coefficients (R² = 0.91–0.94), indicative of a diffusion-controlled process, the superior fit of the Korsmeyer-Peppas model confirms that drug release from the nanofiber system is not solely diffusion-driven. Instead, the release mechanism involves a synergistic interplay of matrix swelling, structural relaxation, and controlled diffusion [39, 40]. Collectively, these findings indicate that Cm-Gel′: Cm-CNC′ nanofibers exhibit a controlled, site-specific drug release profile, characterized by minimal release in the upper gastrointestinal tract and enhanced release in the colon, particularly in response to enzymatic stimuli.

Based on these optimal loading characteristics and its favorable sulfatase- and pH-triggered release behavior, the UV-crosslinked Cm-Gel’: Cm-CNC’ NF loaded with 0.5% 5-ASA was considered the most suitable formulation for oral colon-targeted delivery and was used in cellular and in vivo animal studies.

### Cellular studies

#### Cell viability

The biocompatibility of Cm-Gel NF and Cm-Gel’: Cm-CNC’ NF was evaluated on RAW 264.7 and CaCO-2 cells using WST assay via both extract and direct contact methods (Fig. 6A&B). In extract tests, both nanofibers maintained > 90% viability in both cell lines, indicating minimal cytotoxicity. In direct-contact tests, a mild reduction in viability was observed, Cm-Gel NF showed ~ 80% and ~ 82% viability in RAW 264.7 and CaCO-2 cells, respectively, while Cm-Gel’: Cm-CNC’ NF maintained ~ 90% and ~ 85% viability. The slight reduction might be attributed to the coumaroyl modification of gelatin. Nonetheless, the incorporation of Cm-CNC did not adversely affect the outcome, confirming its suitability for safe drug delivery. The cytotoxicity of 5-ASA and 5-ASA@Cm-Gel’: Cm-CNC’ NF was also compared using RAW 264.7 cells. At 0.25%, 0.5%, and 1.0% 5-ASA concentrations, free drug exhibited significant cytotoxicity (~ 79%, 75%, and 40% viability, respectively). In contrast, cells treated with encapsulated 5-ASA maintained > 80% viability at all concentrations, demonstrating reduced cytotoxicity of the nanofiber formulation due to controlled release and protective encapsulation (Fig. 6E). This confirms that the nanofiber-based system is safe for normal cells, supporting its biocompatibility for oral administration.

#### Cellular uptake

Cellular uptake of calcein-loaded nanofibers was monitored by confocal microscopy in Raw 264.7 cells over 1 h and 4 h (Fig. 6 C &D). Free calcein showed strong initial fluorescence, which diminished by 4 h, indicating rapid diffusion. Cm-Gel NF showed low and relatively constant fluorescence intensity, reflecting limited dye release. In contrast, Cm-Gel’: Cm-CNC’ NF exhibited higher fluorescence, especially after 4 h, suggesting greater release due to disintegration of electrostatic interactions between Cm-Gel and Cm-CNC in the neutral pH of the medium. This supports improved release kinetics and cellular internalization in the CNC-complexed system.

#### Intracellular localization of nanofibers by LAMP1and LysoTracker Green staining

The intracellular trafficking of RBITC-labeled Cm-Gel′: Cm-CNC′ nanofibers was investigated using LAMP1 immunostaining to identify late endosomal and lysosomal compartments (Fig. 7 A). At 6 h, moderate LAMP1-associated fluorescence was detected, indicating initial localization of the nanofibers within late endocytic vesicles following endocytic uptake. By 12 h, a marked increase in LAMP1 fluorescence intensity was observed, reflecting enhanced accumulation and maturation of late endosomal/lysosomal compartments over time. Quantitative fluorescence analysis further confirmed this observation, showing a significant increase in LAMP1-associated fluorescence intensity at 12 h compared with 6 h (Fig. S10A), indicating progressive accumulation of the nanofibers within late endosomal/lysosomal compartments over time. The statistical increase in LAMP1 signal supports the confocal observations and provides quantitative evidence for sustained intracellular trafficking along the endo-lysosomal pathway. This temporal increase suggests progressive transport of the nanofibers along the endo-lysosomal pathway and sustained vesicular processing. It is acknowledged that identification of intracellular compartments may vary depending on cell type and the markers employed [76]. In this study, LAMP1 staining was utilized as a reliable marker for late endosomal and lysosomal compartments, which represent the primary destinations for endocytosed nanomaterials. The observed time-dependent increase in LAMP1-associated fluorescence supports the conclusion that the nanofibers accumulate progressively within these compartments. While LAMP1 distribution may differ among cell types, the consistent increase in fluorescence intensity over time indicates authentic intracellular trafficking rather than experimental artifacts [77]. These findings reinforce the conclusion that Cm-Gel′: Cm-CNC′ nanofibers undergo regulated endo-lysosomal processing, a process that is essential for sustained intracellular drug delivery.

Complementary analysis using LysoTracker Green staining demonstrated a time-dependent modulation of lysosomal activity (Fig. 7B). At 6 h, a decrease in LysoTracker intensity relative to the control was observed, indicating a transient reduction in lysosomal acidification following nanofiber internalization. This early perturbation aligns with known nanoparticle-lysosome interactions, where internalized materials can temporarily affect lysosomal pH or proton pump function. By 12 h, LysoTracker fluorescence intensity increased, reflecting recovery of lysosomal acidity and restoration of normal lysosomal function. This trend was further supported by quantitative fluorescence intensity measurements (Fig. S10B), which revealed significantly higher LysoTracker-associated fluorescence at 12 h than at 6 h. These results suggest that the transient reduction in lysosomal acidification observed during the early stages of nanofiber internalization was followed by recovery of lysosomal activity, indicating successful cellular adaptation and continued intracellular processing of the nanofibers.

Taken together, the combined observations from both markers reveal a dynamic pattern of intracellular processing. The elevated LAMP1 signal at 12 h, alongside the restored LysoTracker intensity, suggests enhanced formation and maturation of lysosomal compartments coupled with the reestablishment of their acidic environment. This indicates that the nanofibers undergo regulated trafficking through the endo-lysosomal pathway, causing only temporary lysosomal disturbance followed by cellular adaptation. Unlike systems that cause prolonged lysosomal dysfunction or rupture, this behavior reflects a biocompatible intracellular processing profile favorable for sustained intracellular drug delivery. Moreover, the progressive increase in LAMP1-positive compartments implies efficient trafficking of nanofibers from early endosomes to late endosomes and lysosomes, rather than rapid recycling or expulsion. This controlled intracellular routing may contribute to prolonged retention of the encapsulated drug, thereby enhancing therapeutic effectiveness.

#### Intestinal epithelial barrier function using transwell monolayer CaCO-2 cell permeability assay

The restoration of intestinal epithelial barrier function was assessed using a CaCO-2 monolayer permeability assay (Fig. 7 C). DSS treatment significantly increased FITC-dextran permeability relative to the untreated control (*p* < 0.001), reflecting compromised epithelial integrity. Treatment with free 5-ASA partially decreased permeability, whereas the 5-ASA-loaded nanofiber group exhibited a significantly greater reduction in FITC-dextran transport (*p* < 0.01 versus free drug), indicating superior barrier restoration. The blank nanofiber group also showed moderate protective effects. This enhanced barrier function is attributed to sustained drug release, improved cellular uptake, and prolonged interaction of the nanofibers with the epithelial surface. In summary, the 5-ASA loaded Cm-Gel′: Cm-CNC′ nanofibers demonstrate efficient intracellular trafficking with time-dependent lysosomal adaptation, significant attenuation of oxidative stress, and effective restoration of epithelial barrier integrity, underscoring their multifunctional therapeutic potential.

#### DCFH-DA assay of nanofibers

Intracellular reactive oxygen species (ROS) levels were quantified using the DCFH-DA assay via flow cytometry (FACS) (Fig. 7D). Lipopolysaccharide (LPS) stimulation induced a significant increase in ROS production compared to the control group (*p* < 0.001). While treatment with free 5-ASA reduced ROS levels, the 5-ASA-loaded nanofiber group achieved a significantly greater reduction (*p* < 0.01 versus free 5-ASA), indicating enhanced intracellular delivery and sustained antioxidant effects. Additionally, the blank nanofiber group demonstrated a moderate decrease in ROS, suggesting an intrinsic protective effect of the nanofiber matrix itself. These results confirm that the nanofiber system effectively mitigates oxidative stress, a major factor in intestinal inflammation [78].

#### Cytokine suppression

The anti-inflammatory potential of the nanofibers was evaluated by measuring TNF-α and IL-6 levels in DSS-induced CaCO-2 cells. DSS treatment significantly elevated IL-6 (~ 140 pg/mL) (Fig. 7E) and TNF-α (~ 700 pg/mL) (Fig. 7 F). Treatment with 5-ASA@Cm-Gel’: Cm-CNC’ NF markedly reduced cytokine levels, nearly restoring them to basal levels observed in control. Nanofiber alone (Cm-Gel’: Cm-CNC’) showed slight but statistically significant suppression of cytokines compared to the DSS-only group. This mild reduction may be attributed to the intrinsic anti-inflammatory properties of coumaric acid, gelatin, and CNC, which have been previously reported to inhibit oxidative stress and inflammatory signaling. Overall, cellular results demonstrate that the developed nanofiber is safe, effectively internalized, and therapeutically beneficial. These findings support its potential for ulcerative colitis treatment through oral delivery of 5-ASA in a colon-targeted and inflammation-responsive manner.

While the CaCO-2 model is widely used to evaluate intestinal barrier function and epithelial inflammatory responses, it does not fully represent the complexity of ulcerative colitis. Conventional Caco-2 monocultures lack immune cells, goblet cells, and microbiota-derived signaling, which are important contributors to intestinal inflammation. Therefore, the observed cytokine modulation primarily reflects epithelial responses and should be interpreted alongside the DSS-induced colitis model, which provides a more physiologically relevant assessment of anti-inflammatory efficacy.

### In vivo biodistribution and targeting efficiency

The colon-targeting ability and biodistribution of DiR-labeled Cm-Gel’-Cm-CNC’ nanofibers (DiR@Cm-Gel’: Cm-CNC’ NF) were evaluated using an IVIS imaging system in DSS-induced colitis mice. After inducing colitis with DSS for 6 days, mice received either free DiR or DiR@Cm-Gel’: Cm-CNC’ NF via oral administration. In vivo imaging showed that free DiR fluorescence declined over 1–8 h, indicating rapid clearance. In contrast, DiR-loaded nanofibers displayed a time-dependent increase in fluorescence intensity, suggesting mucoadhesive retention in the inflamed colon (Fig. 8A&B). Ex vivo imaging of excised colon tissues validated this trend, showing significantly higher fluorescence in the DSS-induced group treated with DiR-loaded nanofibers compared to free dye (Fig. 8 C & E). The elevated retention is attributed to the mucoadhesive nature of gelatin and CNC, which interact preferentially with the inflamed and dysregulated epithelium. Mucoadhesion of gelatin- and cellulose-based biomaterials has been reported to enhance drug retention and localization in gastrointestinal mucosa [79, 79–81]. Furthermore, 24-h ex vivo fluorescence analysis revealed continued signal intensity in the DiR-loaded nanofiber group, suggesting prolonged residence and sustained release (Fig. 8D&F). This behavior is enabled by the nanofiber’s stimuli-responsive properties, sulfatase-induced cleavage of the sulfate group from Cm-CNC and pH-triggered swelling, offering controlled dye release specifically in the disease microenvironment.

In vivo and ex vivo imaging at 8 h post-administration revealed a distinct difference in fluorescence intensity between the DSS and control groups fed with DiR@Cm-Gel′:Cm-CNC′ nanofibers (Fig. 8G-J). The DSS group showed significantly higher fluorescence accumulation in the colon, attributed to the disrupted epithelial architecture caused by colitis, which enhanced nanofiber adhesion and infiltration [4, 6]. Furthermore, the inflamed colon exhibited elevated sulfatase enzyme levels and a lower pH environment, both of which triggered the coumaroyl cleavage and swelling of the nanofiber, promoting targeted and sustained release of the fluorescent dye [19, 66]. In contrast, the control group, with an intact and healthy mucosal barrier, exhibited minimal fluorescence due to limited adhesion, reduced enzymatic activity, and faster clearance via normal peristalsis. These findings confirm that the pathological environment of DSS-induced colitis enhances nanofiber retention and activation, thereby enabling more efficient and localized payload delivery.

### Therapeutic efficacy in DSS-induced colitis model

The therapeutic efficacy of the 5-ASA-loaded, dual-responsive orally administrable nanofiber (5-ASA@Cm-Gel’: Cm-CNC’ NF) was evaluated using a DSS-induced murine colitis model, which closely mimics human UC. Mice were randomly assigned into five groups: (1) Healthy Control, (2) DSS-induced colitis, (3) DSS + Blank Nanofiber (Cm-Gel’: Cm-CNC’ NF), (4) DSS + Free 5-ASA, and (5) DSS + 5-ASA@Cm-Gel’: Cm-CNC’ NF. Colitis was induced by administering 3% DSS in drinking water for 7 consecutive days. Therapeutic interventions (5 mg/kg of free or encapsulated 5-ASA) were administered orally on alternate days, specifically on days 8, 10, 12, and 14.

**Fig. 9:**
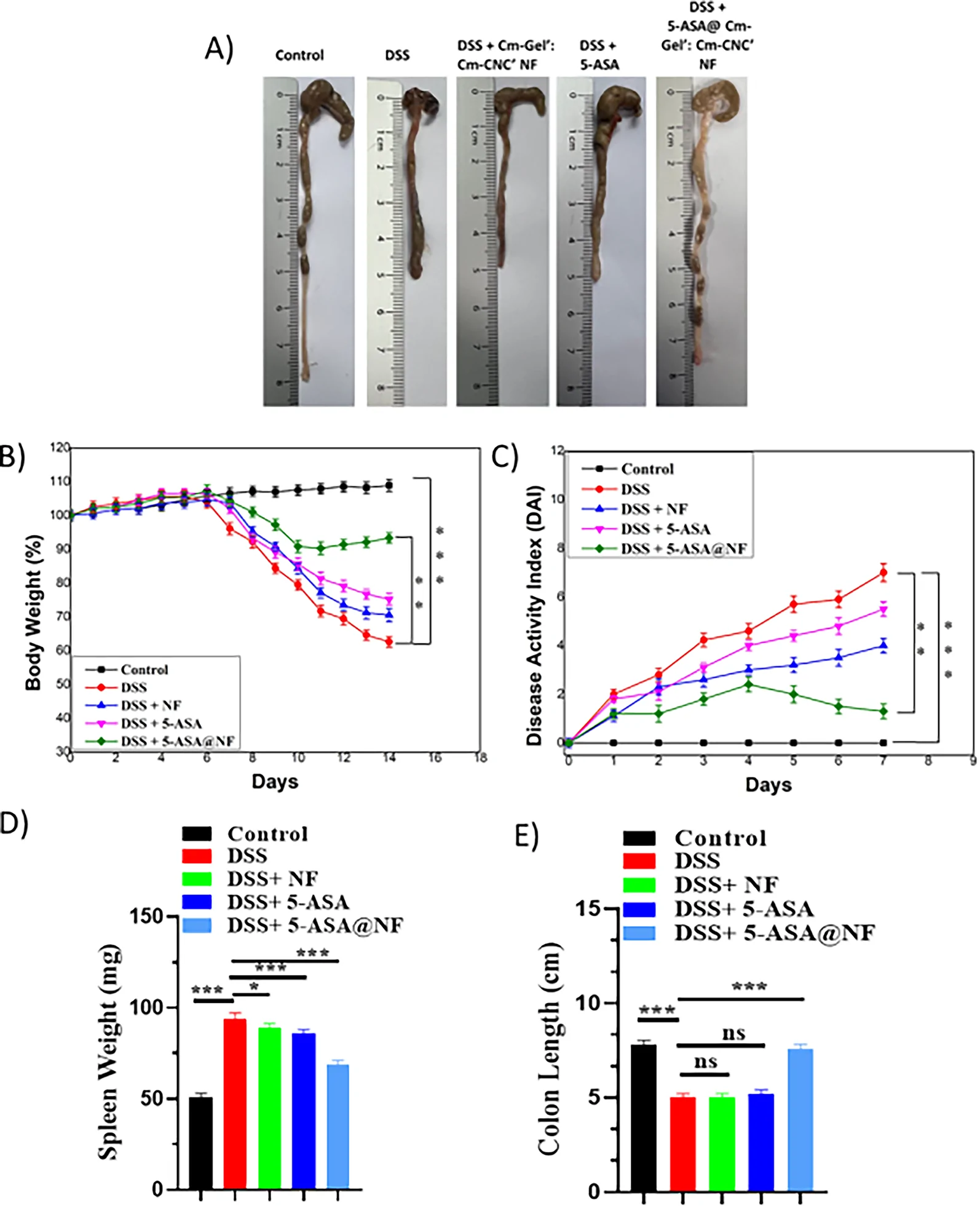

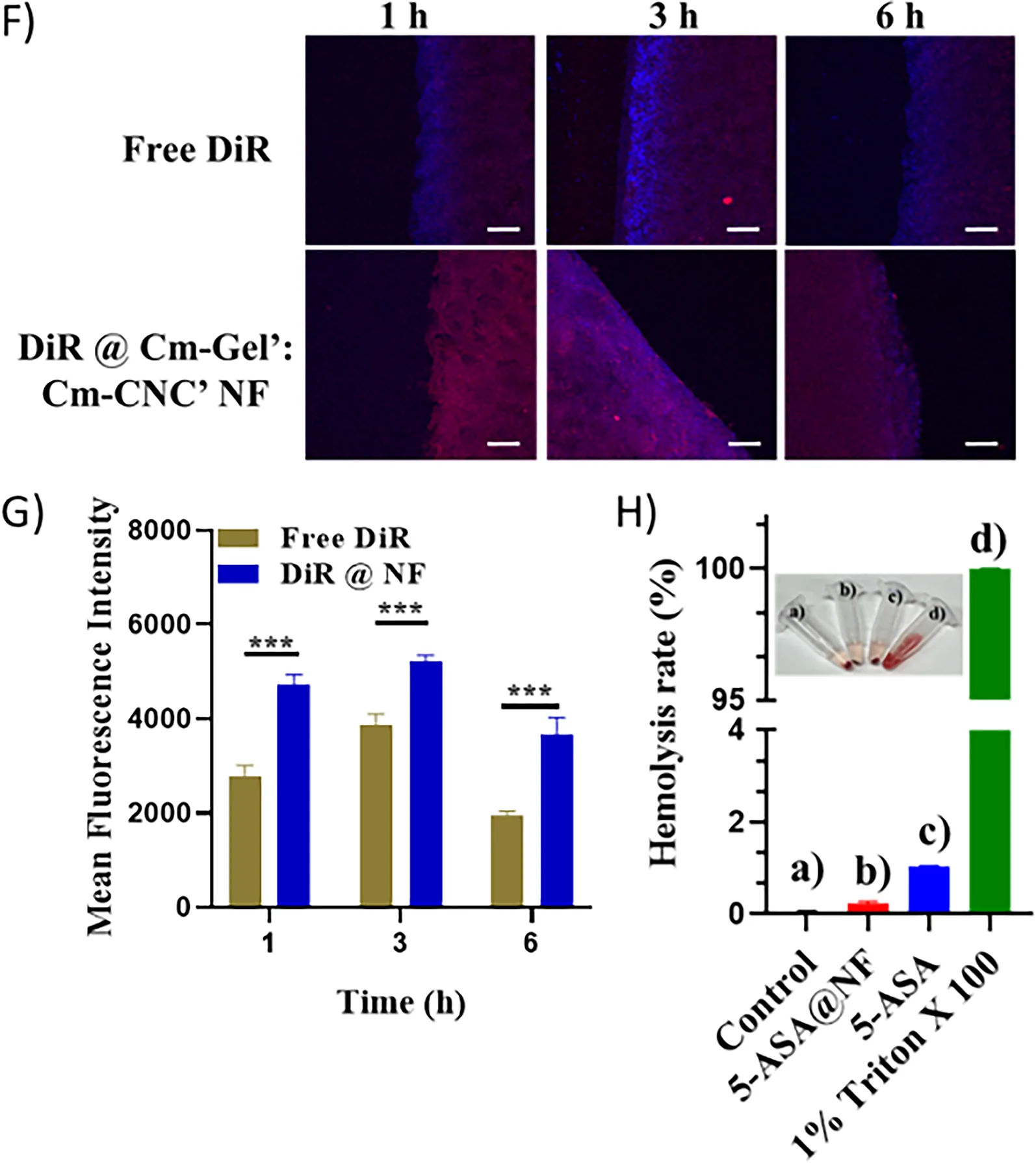
Pharmacodynamic studies of the 5-ASA loaded Cm-Gel’: Cm-CNC’ NF in Ulcerative colitis. **A**) Representative images of the colons after mice were euthanised. (**B**-**E**) Representative images of the body weight loss percentage, Disease Activity Index (DAI), Colon length, and Spleen weight changes in the different groups were analysed. Statistical analysis was performed using one-way ANOVA with Tukey’s multiple comparison test, and data were expressed as the mean ± SD.*p < 0.05, **p < 0.001, and ***p < 0.001 compared with the DSS Group. (**F**) Confocal images showing ex vivo retention and penetration of free DiR and DiR-loaded Cm-Gel′: Cm-CNC′ nanofibers on mouse colon tissues after 1, 3, and 6 h, demonstrating enhanced mucosal adhesion of the nanofiber formulation. (Scale bar − 50 μm). (**G**) Quantitative fluorescence intensity analysis confirms significantly higher colonic retention of nanofiber-loaded DiR compared to free DiR at all-time points. Data are expressed as mean ± SD (*n* = 3). (NF represents Cm-Gel’: Cm-CNC’ NF) Statistical significance was analyzed using one-way ANOVA in GraphPad Prism, with ***p < 0.001 compared to free DiR group. (**H**) Hemolysis assay showing negligible hemolysis for free drug and 5-ASA-loaded nanofibers compared with the Triton X-100 positive control

Throughout the experimental timeline, multiple clinical indicators were monitored, including body weight change, Disease Activity Index (DAI), stool consistency, and rectal bleeding. DSS-treated mice exhibited significant weight loss, elevated DAI scores, and clinical symptoms, including diarrhea and hematochezia, all consistent with acute colitis pathology (Fig. 9A-E). Notably, treatment with 5-ASA@Cm-Gel’: Cm-CNC’ NF significantly attenuated body weight loss compared to both DSS-only and DSS + free 5-ASA groups. The improvement in weight maintenance is attributed to the reduction of systemic inflammation, preservation of colonic epithelial function, and restoration of nutrient absorption (Fig. 9B). These findings align with previous reports linking DSS-induced catabolic stress and mucosal damage to rapid weight reduction in colitis models [4, 82, 83]. Colon length, another well-established indicator of colonic inflammation, was significantly shortened in the DSS group, reflecting tissue contraction and fibrosis due to prolonged inflammation (Fig. 9A&D). However, mice treated with the 5-ASA-loaded nanofiber displayed substantially preserved colon length, surpassing the protective effects observed in both the free drug and blank nanofiber groups. This suggests that the combined effects of localized drug delivery, mucoadhesion, and enzyme/pH-responsive release contribute to improved therapeutic outcomes. In the DSS group, a significant increase in spleen weight was observed, indicating systemic inflammation and immune activation associated with colitis. Treatment with 5-ASA and especially with 5-ASA@Cm-Gel′: Cm-CNC′ NF effectively reduced spleen enlargement, restoring values closer to the control group and suggesting anti-inflammatory efficacy (Fig. 9E). Furthermore, stool consistency and rectal bleeding, hallmark indicators of UC severity, showed marked differences among groups. The DSS group presented with frequent watery stools and visible rectal bleeding (Fig. S11), characteristic of mucosal ulceration and epithelial erosion. In contrast, free 5-ASA treatment moderately improved stool consistency and reduced bleeding, but traces of inflammation remained evident. Remarkably, the 5-ASA@Cm-Gel’: Cm-CNC’ NF group exhibited near-complete normalization of stool appearance and consistency, with no observable signs of rectal bleeding, demonstrating superior mucosal protection and restoration of gastrointestinal function.

Importantly, these therapeutic effects were consistent across all evaluated parameters and closely mirrored those observed in the healthy control group. The convergence of positive outcomes recovered body weight, normalized colon length, and elimination of colitis symptoms, strongly supports the efficacy of the nanofiber-based drug delivery system. These results also reinforce the hypothesis that the enhanced performance of 5-ASA@Cm-Gel’: Cm-CNC’ NF is due to its ability to (i) selectively release the drug in the inflamed colon, (ii) remain localized at the mucosal surface via mucoadhesive interactions, and (iii) sustain therapeutic levels over time due to slow enzymatic degradation and controlled pH response. Overall, the nanofiber-encapsulated 5-ASA not only demonstrated superior therapeutic efficacy compared to free drug administration but also effectively reversed UC-associated physiological and clinical symptoms, highlighting its potential as an advanced, colon-targeted, oral drug delivery platform for long-term UC management.

### Mucoadhesive retention and hemocompatibility evaluation of nanofibers

The mucoadhesive properties ability of Cm-Gel’: Cm-CNC’ nanofibers were assessed using freshly excised mouse colon tissues (Fig. 9 F). Confocal microscopy revealed that free DiR displayed weak fluorescence that rapidly diminished over time, indicating limited retention on the mucosal surface. In contrast, DiR-loaded Cm-Gel’: Cm-CNC’ nanofibers exhibited significantly higher fluorescence intensity at all examined time points (1, 3, and 6 h), demonstrating enhanced mucosal adhesion. Quantitative analysis confirmed that fluorescence retention in the nanofiber group was significantly greater than that of free DiR (*p* < 0.01) at each time interval.

Beyond surface adhesion, the nanofiber formulation showed improved penetration of dye into the mucus layer, as evidenced by a stronger and more evenly distributed fluorescence signal throughout the mucosal depth (Fig. 9G). The sustained fluorescence increase from 1 to 6 h suggests prolonged interaction with the mucus, likely due to the hydrophilic and functionalized polymer network facilitating hydrogen bonding and physical entanglement with mucin glycoproteins.while the hydrophilic Cm-CNC component and porous electrospun architecture promote hydration and diffusion within the mucus network This enhanced retention and penetration are beneficial for colon-targeted drug delivery by promoting localized drug accumulation at inflamed sites. These interactions reduce rapid clearance from the colonic surface and support sustained drug release within the local environment. Overall, the nanofiber system exhibits superior mucosal retention compared to free dye, highlighting its potential for enhanced therapeutic efficacy in ulcerative colitis.

Hemocompatibility of the nanofiber system was evaluated via a hemolysis assay (Fig. 9H). The positive control group treated with Triton X-100 showed complete hemolysis, whereas the untreated control exhibited negligible hemolysis. Both free 5-ASA and 5-ASA-loaded Cm-Gel’: Cm-CNC’ nanofibers demonstrated minimal hemolytic activity, with hemolysis rates remaining below the acceptable 5% threshold [59]. Notably, the nanofiber formulation exhibited hemolysis levels comparable to or slightly lower than free 5-ASA, indicating that the polymer matrix does not cause damage to red blood cell membranes.

These findings confirm that Cm-Gel’: Cm-CNC’ nanofibers possess excellent hemocompatibility and are safe for systemic exposure. Coupled with their strong mucoadhesive and mucus-penetrating capabilities, this nanofiber system presents an effective and biocompatible platform for oral colon-targeted drug delivery.

### Histological and immunohistochemical evaluation

**Fig. 10:**
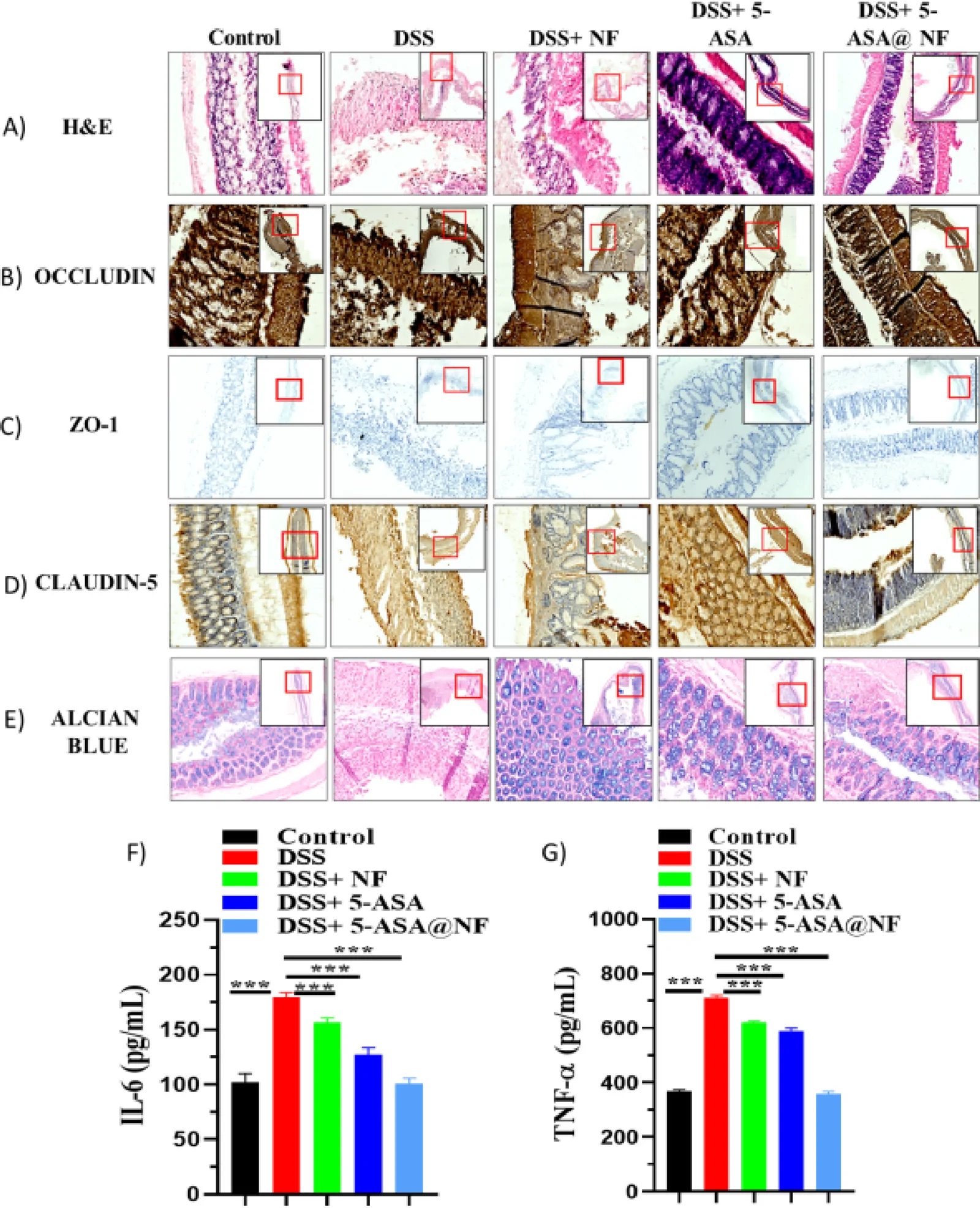
Histological, immunohistochemical staining and cytokine expression studies of DSS-induced Colitis mice after treatment with 5-ASA@ Cm-Gel’: Cm-CNC’ NF treatment. **A**) H&E, **B**) Occludin, **C**) ZO-1, **D**) Claudin-5, and **E**) Alcian blue staining of colon tissues from different groups with full images at the inset is given respectively. (**F**&**G**) Representative image of IL-6 and TNF-α cytokine analysis study conducted using the serum from the blood of the mice of different groups. The data were expressed in mean ± SD (*n* = 3). (NF represents Cm-Gel’: Cm-CNC’ NF) Significance is denoted as *p < 0.05, **p < 0.005, and ***p < 0.001 compared to the DSS group

Histological examination of hematoxylin and eosin (H&E)-stained colon tissues provided clear evidence of the protective effects of 5-ASA-loaded nanofibers in DSS-induced colitis (Fig. 10 A). Colon sections from DSS-treated mice exhibited pronounced pathological alterations, including severe epithelial erosion, crypt architectural distortion, and dense infiltration of inflammatory cells into the mucosal and submucosal layers, hallmarks of acute colitis [1, 4, 60, 82]. Additionally, a significant separation between the mucosal and muscularis layers was observed, which may be attributed to excessive collagen deposition, further indicating tissue remodeling and fibrotic changes. These findings are consistent with previous reports suggesting that DSS-induced colitis disrupts the intestinal epithelial barrier, leading to compromised mucosal integrity and chronic inflammation [6–8]. In contrast, colon tissues from the untreated control group displayed intact and well-organized histoarchitecture, with continuous epithelial linings, healthy crypt structures, and abundant goblet cells. The mucosal and muscularis layers remained closely aligned, with no visible signs of inflammation or tissue damage.

Notably, treatment with 5-ASA@Cm-Gel’: Cm-CNC’ NF substantially preserved colonic structure. Histological features in this group revealed minimal immune cell infiltration, well-defined crypts, and nearly complete restoration of normal tissue morphology. This suggests that the drug-loaded nanofibers not only provided anti-inflammatory protection but also promoted epithelial healing and architectural regeneration. Compared to the free 5-ASA group, which showed moderate improvement with reduced epithelial damage and mild crypt recovery, the nanofiber-encapsulated drug demonstrated significantly superior therapeutic outcomes, likely due to enhanced retention at the inflamed site, prolonged release, and improved mucosal adherence. The group treated with nanofibers alone (without drug) showed partial protection, with observable reductions in immune cell infiltration and structural damage, though these effects were less pronounced than in the 5-ASA-loaded fiber group. This indicates that while the biopolymer matrix itself may exert mild barrier-protective or anti-inflammatory effects, the therapeutic efficacy is greatly enhanced by the incorporation of 5-ASA.

To further validate mucosal protection, immunohistochemical staining was performed to assess the expression and localization of key tight junction (TJ) proteins-Occludin, Claudin-5, and ZO-1, which are essential components of the intestinal epithelial barrier (Fig. 10B-D). DSS exposure led to a marked reduction in expression levels of all three TJ proteins, as well as mislocalization away from the apical membrane, indicating barrier dysfunction and increased mucosal permeability, a defining feature of colitis pathology [8, 60, 84, 85].

In sharp contrast, colon tissues from mice treated with 5-ASA@Cm-Gel’: Cm-CNC’ NF displayed strong, membrane-localized expression of Occludin, Claudin-5, and ZO-1, closely resembling the staining patterns seen in healthy controls (Fig. 10B). This suggests effective restoration of epithelial integrity and tight junction assembly. Compared to the nanofiber-only group, the drug-loaded nanofibers resulted in notably higher expression of TJ proteins, clearly visible through increased staining intensity and uniform distribution across the epithelial surface. While free 5-ASA treatment moderately restored TJ protein expression, it failed to match the robust and consistent re-expression observed in the nanofiber-drug combination group.

Alcian blue staining was employed to evaluate mucus layer integrity in colonic tissues (Fig. 10E). The control group displayed a continuous, intensely stained blue mucus layer, indicative of normal mucin secretion. In contrast, the DSS-treated group exhibited significantly diminished staining, disrupted epithelial architecture, and a loss of goblet cells, confirming pronounced mucus depletion. Treatment with free 5-ASA led to partial restoration of mucin levels; however, the 5-ASA-loaded Cm-Gel′: Cm-CNC′ nanofiber group demonstrated more intense and uniform staining, accompanied by improved epithelial structure and goblet cell regeneration. This enhanced preservation of the mucus layer is attributed to sustained drug release and extended mucosal interaction, which help protect against inflammation-induced mucin loss and facilitate barrier recovery, an essential aspect in the management of ulcerative colitis.

These results strongly support the hypothesis that the dual-responsive, mucoadhesive nanofiber system not only enhances local drug bioavailability but also enables efficient barrier repair by reducing inflammation and preserving epithelial junctional complexes. The data align with prior studies demonstrating that tight junction restoration is a key indicator of mucosal healing in colitis, further justifying the therapeutic potential of the 5-ASA@Cm-Gel’: Cm-CNC’ NF platform for targeted UC treatment.

### Cytokine profiling in colon tissues

To further elucidate the anti-inflammatory effects of the treatment strategies, pro-inflammatory cytokine levels, specifically interleukin-6 (IL-6) and tumor necrosis factor-alpha (TNF-α), were quantified in colon tissue homogenates (Fig. 10 F&G). In the DSS-induced colitis group, both cytokines were markedly elevated, reflecting severe mucosal inflammation and immune activation. This cytokine profile aligns with clinical observations in UC patients, where increased TNF-α and IL-6 are strongly associated with disease severity, epithelial barrier dysfunction, and progressive mucosal damage [11, 84].

Treatment with 5-ASA@Cm-Gel’: Cm-CNC’ NF resulted in a significant reduction in both TNF-α and IL-6 levels compared to the DSS group, demonstrating a strong anti-inflammatory effect. Notably, the nanofiber-based drug delivery system outperformed free 5-ASA, indicating enhanced therapeutic efficacy likely due to the targeted and sustained release of 5-ASA at the site of inflammation. These results underscore the synergistic therapeutic benefit of combining the active pharmaceutical ingredient (5-ASA) with a smart, enzyme- and pH-responsive nanofiber matrix.

The anti-inflammatory effects observed at the cytokine level were consistent with the histological and immunohistochemical findings. Reduced TNF-α levels are particularly significant, as this cytokine plays a pivotal role in compromising tight junction integrity by downregulating epithelial proteins such as Occludin and ZO-1 [84, 86, 87]. In the DSS group, where TNF-α levels were highest, IHC analysis revealed suppressed expression and disrupted membrane localization of Occludin, Claudin-5, and ZO-1, indicative of compromised epithelial barrier function. In contrast, the 5-ASA@Cm-Gel’: Cm-CNC’ NF group showed not only a marked decrease in TNF-α and IL-6 levels, but also a concurrent restoration of tight junction protein expression, confirming that dampening inflammatory signaling contributes to barrier recovery.

This correlation suggests a mechanistic link between cytokine suppression and mucosal healing: reduced TNF-α relieves tight junction protein suppression, thereby promoting reassembly of the epithelial barrier. Similarly, decreased IL-6, a key mediator of chronic inflammation and epithelial apoptosis, contributes to reduced epithelial cell turnover and enhanced mucosal stability. The immunohistochemical evidence of restored junctional integrity in the drug-loaded fiber group thus finds strong biochemical support in the cytokine profile.

Moreover, the superior anti-inflammatory performance of 5-ASA@Cm-Gel’: Cm-CNC’ NF can be attributed to several functional features of the nanofiber system. Its mucoadhesive properties ensure prolonged contact with inflamed mucosa, allowing sustained drug retention and absorption. In addition, the sulfatase- and pH-responsive degradation ensures precise release of 5-ASA specifically in the inflamed colonic environment, where these triggers are overexpressed. This localized release reduces systemic drug exposure and off-target effects, thereby enhancing therapeutic index.

Overall, the significant downregulation of TNF-α and IL-6, coupled with tight junction protein restoration and mucosal healing, demonstrates that the nanofiber formulation not only mitigates inflammation but also promotes epithelial repair. These findings highlight the potential of 5-ASA@Cm-Gel’: Cm-CNC’ NF as a highly effective colon-targeted oral therapy for UC, capable of modulating key inflammatory pathways and restoring barrier function, critical aspects of long-term disease management.

Compared with recently reported colon-targeted delivery systems [3, 33, 34, 36, 37, 88], the Cm-Gel′: Cm-CNC′ nanofiber platform offers several integrated advantages, particularly as an orally administrable nanofiber system capable of enzyme-responsive colonic activation. Unlike conventional single stimuli-responsive carriers, the present formulation provides combitorial pH responsiveness with sulfatase-triggered release, enabling enhanced drug release specifically in the UC physiological environment [88]. In addition to pH-responsiveness, the sulfatase sensitivity is especially advantageous for UC therapy, where microbial enzyme abundance provides a biologically selective trigger for site-specific drug release. Moreover, the as-prepared Cm Gel’: Cm CNC’ NF matrix provides strong mucosal adhesion, prolonged colonic retention (Fig. 9), intracellular trafficking compatible with sustained delivery (Fig. 5), ROS suppression (Fig. 7D), epithelial barrier recovery (Fig. 7 C), and mucus preservation (Fig. 10E). Although recent advanced delivery systems, such as highly specialized targeting molecules-based dual-targeted celecoxib nanoparticles for DSS-induced colitis [9] synthetic polymer–based enteric-glia-targeted oral nanosystems [44], and polysaccharide-based nanoplatforms with strong mucus-penetrating capabilities [47, 89], have demonstrated potent therapeutic effects, the as-synthesized Cm Gel/Cm CNC NF is distinguished by its multifunctional, biocompatible, and fully orally administrable matrix that enables simple dosing while providing sustained, localized drug delivery to the colon. Nevertheless, several limitations remain, including the lack of long-term pharmacokinetic profiling, chronic toxicity assessment, and direct benchmarking against clinically established colon-targeted formulations. Future investigations should aim to fill these gaps, alongside assessing large-scale manufacturing reproducibility and therapeutic efficacy in chronic UC models, to enhance the translational potential of this nanofiber system.

## Conclusion

In this study, we successfully developed an orally administrable, photo-crosslinked nanofiber system composed of coumaroyl-modified gelatin (Cm-Gel) and coumaroyl-modified cellulose nanocrystals (Cm-CNC) for the targeted delivery of 5-aminosalicylic acid (5-ASA) in UC therapy. The nanofiber was fabricated via electrospinning and structurally reinforced through UV-induced coumaroyl dimerization, ensuring mechanical integrity during gastrointestinal transit while preserving enzymatic and pH responsiveness. This dual-responsive platform was engineered to respond specifically to colonic conditions, leveraging elevated sulfatase activity and near-neutral pH for controlled and localized drug release. In vitro studies demonstrated that the nanofiber enabled sustained, on-demand, and site-specific release of 5-ASA, with significantly enhanced drug retention and release kinetics compared to non-crosslinked counterparts. Sequential simulated gastrointestinal release studies further confirmed that the nanofibers remained stable in gastric and intestinal environments while exhibiting accelerated sulfatase-triggered release in simulated colonic fluid, and kinetic modeling revealed diffusion-controlled enzyme-responsive release behavior consistent with Higuchi and Korsmeyer–Peppas mechanisms. Cellular interaction studies confirmed effective uptake of the nanofiber system by colonic epithelial cells, with excellent biocompatibility. Intracellular trafficking analysis using LAMP1 and LysoTracker Green staining demonstrated time-dependent lysosomal localization and recovery of lysosomal activity, confirming regulated endo-lysosomal processing of the nanofibers after cellular internalization. In addition, DCFH-DA flow cytometric analysis revealed significant suppression of intracellular ROS generation, indicating effective oxidative stress reduction by the 5-ASA-loaded nanofiber system. Hemolysis testing further verified excellent hemocompatibility, with negligible red blood cell damage confirming the biosafety of the developed formulation. In vivo validation in a DSS-induced murine colitis model revealed that 5-ASA@Cm-Gel’: Cm-CNC’ NF not only suppressed clinical symptoms such as weight loss, diarrhea, and colon shortening, but also significantly reduced pro-inflammatory cytokines (TNF-α and IL-6) in colon tissues. These biochemical findings were corroborated by histological analyses, where the drug-loaded nanofiber restored colonic architecture and tight junction protein expression (Occludin, Claudin-5, ZO-1), indicating epithelial barrier healing. Collectively, this study highlights the potential of 5-ASA@Cm-Gel’: Cm-CNC’ NF as a next-generation, colon-targeted oral drug delivery platform. Its unique combination of enzymatic and pH sensitivity, mucoadhesion, and structural stability positions it as a promising therapeutic strategy for the effective, localized, and non-invasive treatment of ulcerative colitis.

## Supplementary Information

Supplementary Material 1

## Data Availability

No datasets were generated or analysed during the current study.
