# Supplementary material for: Orally administrable photo-crosslinked coumaroyl-modified gelatin-CNC nanofibers for ulcerative colitis environment-triggered release of 5-aminosalicylic acid
Source: J Nanobiotechnology. 2026 Aug 12;24:1044. doi: 10.1186/s12951-026-04889-y (PMC13746550; doi:10.1186/s12951-026-04889-y)
Supplement: Supplementary file 1 [file 12951_2026_4889_MOESM1_ESM.docx]

**Orally administrable photo-crosslinked Coumaroyl-modified Gelatin-CNC nanofiber for ulcerative colitis environment-triggered release of 5-aminosalicylic acid**

Jomon George Joy^a^, Garima Sharma^b*^, Mydhili Govindarasu^a^, Panalee Pomseethong^a^, Songrae Kim^c^, and Jin-Chul Kim^a,*^

^a^ Department of Biomedical Science & Institute of Bioscience and Biotechnology, Kangwon National University, Chuncheon 24341, Republic of Korea

^b^ Institute of Forest Science, Kangwon National University, Chuncheon, 24341, Republic of Korea

^c^ Metropolitan Seoul Center Korea Basic Science Institute (KBSI) Seoul 02841, Republic of Korea

Corresponding authors at: ^a^ Department of Biomedical Science & Institute of Bioscience and Biotechnology, Kangwon National University, Chuncheon 24341, Republic of Korea

^b^ Institute of Forest Science, Kangwon National University, Chuncheon, 24341, Republic of Korea

^*^Corresponding authors: [jinkim@kangwon.ac.kr](mailto:jinkim@kangwon.ac.kr) (J.-C. Kim), Tel.: +82 33 250 6561; Fax.: +82 33 259 5645, [microbio.garima@gmail.com](mailto:microbio.garima@gmail.com) (G.S.)

**Supplementary Data**

Fig. S1. 1H-NMR spectrum of A) Gelatin, B) Coumaroyl modified gelatin (Cm-Gel).

Fig. S2. 1H-NMR spectrum of A) Coumaric acid, B) CNC, and C) Cm-CNC (3-1).

Fig. S3. A) FT-IR Spectrum of Gelatin and Cm-Gel. B) FT-IR spectrum of CNC and Cm-CNC with different molar ratios. C) Degree of dimerization of Cm-Gel and Cm-CNC for the confirmation of the photodimerization analysis.

Fig. S4. A) Elemental analysis of carbon and oxygen atom percentage from Cm-Gel: Cm-CNC (9:1) Nanofiber and B) Cm-Gel: Cm-CNC (8:2) nanofiber, respectively (Scale-5 µm)

Fig. S5. A) Confocal image of nanofibers Nile red, a hydrophobic dye, loaded in the nanofibers of Cm-Gel: Cm-CNC (8.6:1.4) mass ratio, B) RBITC, a hydrophilic dye, loaded in the nanofibers of Cm-Gel: Cm-CNC (8.6:1.4).

Fig. S6. A) UV spectrum of different concentrations of the 5-ASA B) Standard curve obtained from the 5-ASA UV spectrum

Fig. S7. Long-term stability of Cm-Gel: Cm-CNC (8:2) nanofibers after 6 months of storage. SEM images of (A) 6-month stored and (B) freshly prepared nanofibers showing preserved fibrous morphology without significant structural changes. (C) Stress–strain curves demonstrating maintained mechanical integrity of stored Cm-Gel: Cm-CNC (8:2) nanofibers compared with Cm-Gel. Scale bar = 1 μm.

Fig. S8. Morphological stability of Cm-Gel′: Cm-CNC′ NF under simulated gastrointestinal conditions. SEM images showing sequential structural changes of the nanofibers after incubation in (A) simulated gastric fluid (SGF), (B) simulated intestinal fluid (SIF), and (C) simulated colonic fluid (SCF). The nanofibers maintained their fibrous morphology in SGF, while partial surface erosion and fragmentation (red circles) were observed in SIF and became more pronounced in SCF, indicating progressive swelling and degradation under colonic conditions. Scale bar = 1 μm

Fig. S9. A) Release profile of Calcein dye with various sulfatase enzyme concentration from Cm-Gel’: Cm-CNC’ NF in A) pH 4.5 without UV cross-linking, B) pH 4.5 with UV cross-linking, C) pH 5.5 without UV cross-linking, D) pH 5.5 with UV cross-linking. E) Cumulative release of 5-ASA with and without sulfatase enzyme in simulated gastrointestinal conditions (n=3). Significance is denoted as *p<0.05, **p<0.005, and ***p<0.001.

Fig. S10. Quantitative analysis of intracellular trafficking and lysosomal activity of RBITC-labeled Cm-Gel′: Cm-CNC′ nanofibers in RAW 264.7 cells. (A) Mean LAMP1 fluorescence intensity at 6 and 12 h. (B) Mean LysoTracker fluorescence intensity at 6 and 12 h. Data are presented as mean ± SD (n = 3). *p < 0.05, ***p < 0.001, ns = not significant.

Fig. S11. Representative images from each treated group for the rectal bleeding, which is a symptom of ulcerative colitis.
